# Assessing heavy metal contamination and health risks in playground dust near cement factory: exposure levels in children

**DOI:** 10.1007/s10653-024-02144-7

**Published:** 2024-08-21

**Authors:** Aşkın Birgül

**Affiliations:** Faculty of Engineering and Natural Sciences, Department of Environmental Engineering, Bursa Technical University, Mimar Sinan Mahallesi Mimar Sinan Bulvarı Eflak Caddesi No:177, 16310 Yıldırım/Bursa, Turkey

**Keywords:** Children's playground, Enrichment factor, Geoaccumulation index, Health risk, Heavy metal, Statistical analysis

## Abstract

Heavy metals pose significant threats to human health, particularly children. This study aimed to assess heavy metal pollution in children's playgrounds using surface dust as an indicator and to ascertain the associated exposure levels and health risks. A total of one hundred twenty dust samples were collected from the surface of playground toys in areas surrounding the cement factory in Bursa, Türkiye, on precipitation-free days. The heavy metal content of the samples was analyzed using ICP-MS. The average total concentration of heavy metals ranged from 2401 to 6832 mg/kg across the sampling sites, with the highest values observed at PG4, PG3, PG2, and PG1, respectively. The most commonly detected heavy metals in the samples included Cr, Mn, Fe, Co, Cu, Zn, Pb, Cd, As, and Ni. Statistical analysis revealed significant positive correlations (*p* < 0.01) among Cr, Mn, Fe, Co, Cu, Pb, As, and Ni, with Cu and Pb also showing correlation (*p* < 0.05). PCA analysis identified three principal components explaining 79.905% of the total variance. The Hazard Quotient (HQ) and Hazard Index values for heavy metals were found to be below the safe threshold (HQ < 1). Quantitative techniques such as the geoaccumulation index and enrichment factor are used to determine pollution levels at the sampling sites. Overall, the results indicate that cement factories significantly contribute to heavy metal pollution, with observed values varying based on proximity to the emission source.

**Supplementary Information:**

The online version contains supplementary material available at 10.1007/s10653-024-02144-7.

## Introduction

Particle matter (PM) emissions have significantly grown due to the rapid economic growth and ongoing improvement of people's material lifestyles, which has a detrimental effect on both the environment and human health. The primary components of PM in the atmosphere, which is complicated, include metals, sea salt, soot, fly ash, organic matter, sulfate, and nitrate (Chu et al., 2020; Nie et al., 2022). PM can absorb different heavy metals, altering their chemistry and toxicity in the process. Living in such a setting for an extended period might result in chronic and acute health conditions (Gulia et al., 2019; Heal et al., 2012).

Heavy metals (HMs) are persistent hazardous contaminants with specific gravities greater than 5.0 g/cm^3^ and linked to the contamination of the physical and biological environment and their potential toxicity to living organisms (Haygarth & Jarvis, 2002). The toxicity of HMs depends on their chemical composition, the dose and route of exposure, and the age, gender, genetics, and nutritional status of the exposed individuals (Bhat et al., 2019). HMs are naturally occurring elements in the Earth's crust and can be released through natural processes. However, most HM pollution is typically caused by human activities. Unregulated recycling of waste, mining activities, foundries, industrial emissions, vehicle exhaust, and the combustion of fossil fuels are among the numerous sources contributing to the presence of HMs in the environment (Wang et al., 2018a, 2018b, 2018c; Mohammadi et al., 2019; Moghaddam et al., 2022; Jafarzadeh et al., 2022; Naimabadi et al., 2022). When it enters the environment, HMs can accumulate to harmful levels under various conditions, negatively impacting both the environment and human health (He et al. 2020). While small amounts of certain heavy metals like copper (Cu) and zinc (Zn) are essential for health, others like lead (Pb) and cadmium (Cd) can be harmful even at low concentrations, potentially leading to cancer, cardiovascular diseases, high blood pressure, and severe intellectual disability (Esmaeilzadeh et al., 2019; Li et al., 2016; Vasseghian et al., 2020). Therefore, comprehending HMs and their potential contamination sources is crucial for managing and preventing health risks. Due to the varied impacts of HMs on human health, health risk assessment (HRA)-an integrated approach that evaluates health risks posed to humans through exposure to specific substances in environments-can be employed to estimate the potential risks associated with exposure to these toxic substances (Fallahzadeh et al., 2018; Mohammadi et al. 2019; Alidadi et al., 2019; Esmaeilzadeh et al., 2019; Jafarzadeh et al. 2020; Khalid et al., 2020; He et al. 2020; Jafarzadeh et al., 2022; Naimabadi et al., 2022; Moghaddam et al., 2022). In this study, the carcinogenic and non-carcinogenic risks were estimated using the methodology recommended by USEPA (USEPA, 2001). In recent years, to evaluate the degree of intensity of HM contamination in dust and its ecologic risk, different geochemical indicators have been developed (Yongqiang et al., 2012). The pollution levels of studied HMs in samples were examined by indices like the Enrichment factor (EF) or the Geoaccumulation index (I_geo_) (Esmailzadeh et al. 2019).

The propensity of dust particles to attach to HM increases with particle size. Due to increasing industrialization and urbanization, metropolitan areas currently exhibit significant levels of HM pollution (Johnson et al., 2011; Liu et al., 2019).

Industrial operations are a major cause of pollution, and cement manufacture is one of these industrial activities. A single cement mill releases a significant amount of pollution into the atmosphere. Furthermore, a rise in industrial output, a change in fuel type and usage, and dust control technologies all have an impact on the amount and concentration of contaminants discharged. The cement manufacturing sector has been shown to be the main source of particulate matter in many studies in the literature, and it is stated that 20–30% of industrial emissions, or 40% of total industrial emissions, originate from this sector (Hua et al., 2016; Sánchez-Soberón et al., 2015).

In the literature, several studies found that communities near cement facilities complained about air pollution (Kim et al., 2015; Lee et al., 2016). The high coating of dust on parked automobiles or streets and the polluted environment caused confusion and public outrage among neighbors near the facility in most situations. Most of these folks are ignorant of the risks of cement dust in their surroundings (Adeyanju & Okeke, 2019).

HMs have been found in the air and soil near cement plants (Lv et al., 2018; Wang et al., 2018a, 2018b, 2018c) and in biological samples like blood, urine, and hair (Dong et al., 2015; Hwang et al., 2018). There is little data on the body accumulation of various metals in children who live close to these industrial facilities, even though the pediatric age appears to be particularly susceptible to emissions produced by cement plants (Garcia-Perez et al., 2017; Marcon et al., 2014).

A children's playground is the place where children spend a significant part of their time in their daily lives. Due to their inherent hyperactivity and playfulness, children attract more dust to their faces, hands, clothes, and hair. Children will be exposed to heavy metals from the dust through skin contact, ingestion through the hands, and inhalation through the mouth (De Miguel et al., 2007). The primary risk factor for children's health due to ingestion is their frequent hand-to-mouth contact and physical interaction with their surroundings (Rasmussen et al., 2001; Zeng et al., 2016). This increased exposure to HMs can have severe consequences for their health. Due to their younger age and lower body weight, children are more susceptible to the absorption of these metals by their digestive systems. This increased sensitivity to these metals makes children more vulnerable to their adverse effects than adults (Muhammad et al., 2011). Studying the pollution features and health concerns of HMs in playground surface dust is particularly helpful in light of the aforementioned reasons. HM exposure in children can induce allergic responses, renal damage, digestive issues, and neurodevelopmental abnormalities. Lead exposure causes intellectual development regression in children (Han et al., 2002; WHO, 2006).

There are numerous studies in the literature that examine the presence of HM concentrations in soil samples taken from playgrounds situated in various residential districts and at varying distances from the city center (Gagiu et al., 2015; Gasiorek et al., 2017; Solgi, 2016; Tijhuis et al., 2002; Wang et al., 2018a, 2018b, 2018c; Wei & Yang, 2010; Zhaoyong et al., 2018).

It is necessary to conduct focused studies and monitor the levels of HMs in the dust, especially in playground areas, to protect the safety of children in urban settings. Studies on surveys and monitoring of HMs are available in the literature on this topic in various countries (Johnson et al., 2011). HM pollution studies should emphasize evaluating potential pollution-induced health concerns for children, evaluating dust pollution levels, and identifying likely pollution sources (Lai et al., 2010; Wu et al., 2015).

Although there are numerous studies on HM pollution in dust and urban soils in Türkiye (Guney et al., 2010a; Guven, 2019; Sezgin et al., 2019; Yaylalı-Abanuz, 2011; Yesilkanat & Kobya, 2021), there is limited information on HM contamination in children's playgrounds (Guney et al., 2010b; Parlak et al., 2022; Yesil & Yesil, 2019). Furthermore, to the best of the author's knowledge, no studies have investigated HM concentrations in surface dust from children's playgrounds in Bursa (Türkiye). This study aims to evaluate the concentrations of HMs in surface dust samples from playgrounds in Bursa (Türkiye) and the exposure of children to these HMs. The selected HMs are indicators of environmental contamination due to anthropogenic activities in urban areas (Charlesworth et al., 2011). In this context, HM levels were assessed in surface dust samples collected by wiping filter paper in various playgrounds within a 3.5 km radius of the cement factory in Bursa. A risk assessment was conducted for the HM levels children are exposed to while playing. Additionally, the intensity of HM contamination in this area was measured using geochemical indices and statistical analyses.

## Material and methods

### Study area and sample collection

**Fig. 1:**
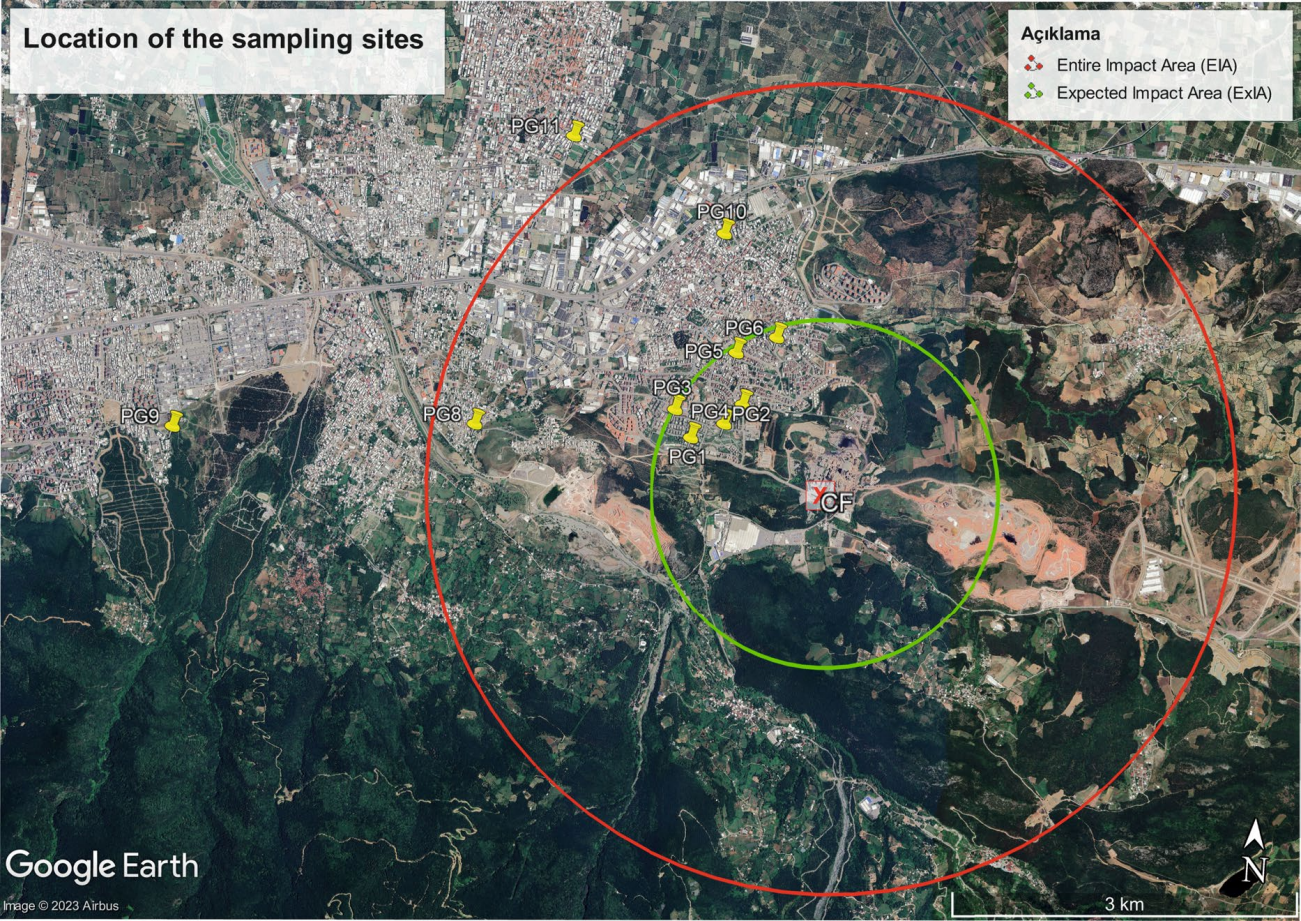
Location of sampling sites

According to the Air Quality Assessment and Management Regulation (MEUCC, 2013), factory-sourced pollutants can have an impact up to a distance corresponding to a radius of 50 times the height of the factory stack. In the same regulation, the maximum stack height in factories is stated as 70 m. In this context, the possible impact area of the cement factory was calculated as a maximum of 3.5 km, and the region within a 3.5 km radius around the cement factory was determined as the entire impact area (EIA). The area within a 1.5 km radius around the cement factory was also determined to be the expected impact area (ExIA). The dust samples to be examined within the scope of this study include surface dust samples taken from playgrounds between 1.5 and 3.5 km around the cement factory. In this context, 11 children’s playgrounds within the EIA near the cement factory have been selected for sampling in Bursa Province. Bursa, located in the northwest region of Türkiye, is the fourth most populous city in the country, with a population of more than 3 million residents and several structured industrial zones housing the automotive, textile, and food sectors. The locations of the selected playgrounds are shown in Fig. 1, and the playgrounds' characteristics are given in Table 1.

**Table 1:** Characteristics of playgrounds

Playgrounds	Distance of the PGs (km) and direction relative to the cement factory	Coordinates
PG1	1.18 km NW	40^o^11′14.15″N, 29^o^12′37.48″E
PG2	0.95 km NW	40^o^11′18.57″N, 29^o^12′49.25″E
PG3	1.39 km NW	40^o^11′21.68″N, 29^o^12′31.06″E
PG4	0.92 km NW	40^o^11′24.26″N, 29^o^12′55.51″E
PG5	1.27 km NW	40^o^11′38.49″N, 29^o^12′52.00″E
PG6	1.27 km NW	40^o^11′43.45″N, 29^o^13′06.02″E
PG7	12.4 km W	40^o^17′24.26″N, 29^o^08′01.98″E
PG8	2.99 km W	40^o^11′14.02″N, 29^o^11′19.09″E
PG9	5.66 km W	40^o^11′07.87″N, 29^o^09′27.13″E
PG10	2.27 km NW	40^o^12′11.64″N, 29^o^12′45.39″E
PG11	3.65 km NW	40^o^12′35.97″N, 29^o^11′47.10″E

Glass fiber filters (GFFs) are used to collect dust samples from the surface of the playground’s toys. GFFs were baked at 450 °C overnight to prevent possible contamination. After cooling to room temperature in the desiccator, it was weighed and packed in pre-baked aluminum foil and then stored at − 20 °C before use. Dust on toys in each playground was collected by wiping with a GFF moistened with acetone. After the sampling, the GFFs were returned to the pre-heated aluminum foil, put in a sealed plastic bag, and stored at a temperature of − 20 °C until the analysis. In total, 120 samples were collected between October 2022 and March 2023, on days that did not experience any rainfall.

### Chemical analysis

Digestion of heavy metals from dust samples was carried out by a hot plate-open vessel procedure. In this procedure, filters used to collect surface dust samples were digested in an acid mixture of HNO_3_:HF (3:2) and boiled at 150 °C in covered beakers on a hot plate for 30 min under the fume cupboard (Geana et al., 2011). After collection, the samples were chilled and passed through a 45 µm pore filter. Afterward, the tubes and filters were rinsed, and the filtrate was diluted to a 50 ml volume with ultra-pure water before obtaining the readings. The blanks and standards were prepared in the same way as the samples, with careful dilution.

The method’s accuracy was tested by analyzing a certified reference material (NIST-certified SRM 2583 (Trace Elements in Indoor Dust)) weighing 0.2 g. The relative standard deviation values between the analysis data and the metal concentrations contained in SRM 2583 are shown in Table 2.

**Table 2:** Certified reference material analysis results

	Concentration (mg/kg)		
	As	Cd	Cr
SRM 2583	7.0 ± 1.6	7.3 ± 3.7	80 ± 22
Analysis result	5.8 ± 0.3	7.5 ± 0.5	54 ± 4.1
% Relative standard deviation (RSD)	17.1	3.1	24.7

### Instrumental analysis

The analysis of the samples was conducted using an Agilent 7700 Model Inductively Coupled Plasma-Mass Spectrometer (ICP-MS). To prepare the calibration stock solution for each of the investigated elements, the multi-element standard solutions for ICP-MS (Agilent ICP-MS Stock Tuning Solution 5188–6564) were diluted appropriately. The calibration curves were generated for each metal by using the least squares fitting method. A mixture of Bismuth (Bi), Germanium (Ge), Indium (In), Lithium (Li), Lutetium (Lu), Rhodium (Rh), Scandium (Sc), and Terbium (Tb) was used as an internal standard. The digested solutions were analyzed for the concentrations of seventeen elements, including antimony (Sb), arsenic (As), barium (Ba), beryllium (Be), cadmium (Cd), chromium (Cr), cobalt (Co), copper (Cu), cyanide (Sn), iron (Fe), lead (Pb), manganese (Mn), nickel (Ni), strontium (Sr), thallium (Tl), vanadium (V) and zinc (Zn). The calibration curves' r^2^ values in the concentration ranges of 1–100 ppb and 1–500 ppb are presented in Table 3. The ICP-MS general operating parameters were as follows: integration time of 0.1 s, sampling time of 0.31 s, data acquisition time of 22.76 s, RF power of 1550 W, RF voltage of 1.78 V, carrier gas flow of 0.9 L/min, make-up gas flow of 0.1 L/min, nebulizer pump rate of 0.1 revolutions per second (rps), and gas flow of 4.5 mL/min. The measurements were carried out in triplicate.

**Table 3:** r^2^ values of the multi-element calibration standard with ICP-MS

HMs	Cd	Pb	V	Cr	Mn	Co	Ni	Cu	Zn	As
1–100 ppb	0.99991	0.99939	0.99918	0.99889	0.99990	0.99956	0.99991	0.99814	0.99980	0.99988
1–500 ppb	0.99991	0.99767	0.99992	0.99978	0.99981	0.99980	0.99968	0.99965	0.99920	0.99982

### Quality assurance (QA)/quality control (QC)

Before use, all equipment utilized for sample preparation was cleaned with 15% nitric acid (HNO_3_) and ultra-pure water three times. The reagents used in the experiment were all of ultra-pure quality.

The Instrument Detection Limit (IDL) refers to the minimum concentration of an analyte discernible from background noise by a specific instrument. At the same time, the Method Detection Limit (MDL) signifies the lowest concentration of a substance measurable and reportable with 99% confidence that the analyte concentration exceeds zero. Calculations for IDL and MDL were performed as follows: IDL equals the lowest calibration level at which a signal is distinguishable from a reagent blank at a 3:1 signal-to-noise (S/N) ratio; additionally, IDL equals MDL if the analyte is absent in the blank sample. MDL is determined as the average concentration of the target chemical in the blank plus three times the standard deviation (WDNRL 1996). Table 4 provides a comprehensive list of MDLs for target chemicals.

**Table 4:** IDL and MDL values of HMs

HMs	IDL	MDL
Be	0.021	0.021
V	0.021	0.654
Cr	0.021	0.391
Mn	0.021	0.383
Fe	0.021	11.380
Co	0.021	0.022
Ni	0.021	7.832
Cu	0.206	0.651
Zn	0.021	8.030
As	0.021	0.121
Se	0.021	1.054
Sr	0.021	0.641
Cd	0.021	0.026
Sn	0.021	0.082
Sb	0.021	0.154
Ba	0.041	0.210
Tl	0.021	0.021
Pb	0.021	0.047

The ICP-MS's sample injection system consists of a temperature-controlled nebulizer and spray chamber connected to an auto-sampler. By maintaining consistent operating conditions throughout the measurement period, the ICP-MS's responsiveness was maintained. All samples were prepared and analyzed, and results were reported as the mean with a 95% level of confidence to ensure repeatability.

### Human exposure and health risk assessment

Health risk assessment involves evaluating the possible health impacts on humans resulting from exposure to environmental pollutants. The United States Environmental Protection Agency (USEPA) has delineated four fundamental stages for this assessment, which encompass hazard identification, establishing dose–response relationships, evaluating exposure levels, and characterizing risks (USEPA, 2001). In this study, the non-carcinogenic health risks related to exposure to HMs through inhalation, ingestion, and skin contact were investigated.

#### Non‑carcinogenic health risk

HM exposure in humans occurs primarily by inhalation, ingestion, and skin contact (Khan et al., 2016). In this study, non-carcinogenic health hazards from exposure to HMs were evaluated using the hazard quotient (HQ) and hazard index (HI) supplied by the following formulae (USEPA, 2001);

Human exposure to HMs predominantly happens through breathing, swallowing, and skin interaction (Khan et al., 2016). This study assessed the non-carcinogenic health risks associated with HM exposure by employing the hazard quotient (HQ) and hazard index (HI) formulas provided by the USEPA (2001);

1 $$HQ=\frac{ADD}{RfD}$$

2 $$HI=\sum HQi={HQ}_{inh}+{HQ}_{ing}+{HQ}_{dermal}$$

In this context, the average daily intake of HMs is symbolized as ADD, measured in mg/kg-day, while the reference dose of HMs for ingestion, inhalation, and skin contact is denoted as RfD, also measured in mg/kg-day (USEPA 1997; Chu et al., 2020). The ADD values for HM exposure via inhalation, ingestion, and skin contact can be calculated using Eqs. (3)-(5) recommended by the USEPA (2001).

3 $${ADD}_{inh}=\frac{CxInhRxEFxED}{PEFxBWxAT}$$

4 $${ADD}_{ing}=\frac{CxIngRxEFxED}{BWxAT}x{10}^{-6}$$

5 $${ADD}_{dermal}=\frac{CxSAxAFxABFxEFxED}{BWxAT}x{10}^{-6}$$

The risk assessment parameters used in this study are presented in Table 5, and cancer slope factors (CSFs) and RfDs are displayed in Table 6.

**Table 5:** The risk assessment parameters

Parameter	Definition	Unit	Value		References
C	HM concentration at the sampling sites	mg/m^3^ for conc. in atmosphere and mg/kg for conc. in dust	Children	Adults	Abdulaziz et al. (2022)
InhR	Inhalation rate	m^3^/day	10	20	USEPA (1997)
IngR	Ingestion rate	mg/day	200	100	USEPA (2002)
EF	Exposure frequency	days/year	350	350	USEPA (2002)
ED	Exposure duration	years	6	24	USEPA (2004)
PEF	Particle emission factor	m^3^/kg	1.36 × 10^9^	1.36 × 10^9^	USEPA (2001)
BW	Average body weight	kg	15	70	USEPA (2004)
AT	Average time	days	365 × ED	365 × ED	USEPA (2001)
SA	Surface area of skin	cm^2^	1600	4350	Zheng et al. (2010)
AF	Skin adherence factor	mg/cm^2^	0.2	0.07	USEPA (2004)
ABF	Absorption factor (dermal)	unitless	0.001	0.001	USEPA (2004)

**Table 6:** RfDs of HMs (mg/kg.day) adopted in this study

	Cr	Mn	Fe	Co	Cu	Zn	Pb	Cd	As	Ni	References
RfD_Ing_	3 × 10^–3^	1.4 × 10^–1^	7 × 10^–1^	3 × 10^–4^	4 × 10^–2^	3 × 10^–1^	3.5 × 10^–3^	1 × 10^–3^	3 × 10^–4^	2 × 10^–2^	USEPA (1994); (1997); (2001); (2004); (2011)
RfD_Inh_	2.85 × 10^–5^	5 × 10^–5^	5 × 10^–5^	6 × 10^–6^	1,4 × 10^–3^	3 × 10^–1^	3.5 × 10^–3^	1 × 10^–3^	3 × 10^–4^	2.06 × 10^–2^	USEPA (1994); (1997); (2001); (2004); (2011)
RfD_Der_	5 × 10^–5^	1.4 × 10^–1^	7 × 10^–1^	3 × 10^–4^	1.2 × 10^–2^	6 × 10^–2^	5.25 × 10^–4^	1 × 10^–5^	3 × 10^–4^	5.4 × 10^–3^	USEPA (1994); (1997); (2001); (2004); (2011)
CSF_inh_	4.10 × 10^1^			9.8			4.2 × 10^–2^	6.1	1.5	8.4 × 10^–1^	USEPA (1994); (1997); (2001); (2004); (2011)

If the HQ value falls below 1, it indicates minimal chances of adverse health effects through a specific exposure route. Conversely, an HQ exceeding 1 suggests a significant likelihood of health risks linked to hazardous materials (HMs) exposure within the studied age group and areas (USEPA, 2001). A HI below 1 signifies a negligible risk of non-carcinogenic health effects. Conversely, an HI exceeding 1 indicates an increased probability of such effects, with the likelihood escalating as the HI value increases.

#### Incremental lifetime cancer risk (ILCR)

As per the classification by the International Agency for Research on Cancer (IARC), cadmium (Cd), hexavalent chromium (Cr (VI)), arsenic (As), and nickel (Ni) are designated as carcinogenic agents (IARC, 2011). This study assessed the cancer risk linked to inhaling these carcinogens throughout an individual's lifetime, employing Eq. (6).

6 $$ILCR=ADD x CSF$$

The incremental lifetime risk is denoted as ILCR, with CSF representing the cancer slope factor, measured in mg/kg/day. The CSF data for carcinogenic hazardous materials were sourced from the California Office of Environmental Health Hazard Assessment (OEHHA 2011). According to the USEPA, an ILCR value below 10^–6^ is considered acceptable, while the cumulative ILCR for multiple carcinogens should not exceed 10^–4^ (Chalvatzaki et al., 2019).

### Geochemical indices

Geochemical indices (GCIs) are defined as the ratio of element concentrations in a potentially hazardous environment to their background reference levels. GCIs are utilized to assess the physical and chemical changes in soil over geological time (Heidari et al., 2022). A common method for evaluating soil contamination in environmental studies involves comparing the contaminant levels at a study site with a reference value (Wu et al., 2015). Since metal particles from both natural and human sources accumulate in the environment (Loring, 1991), it is essential to separate these sources (Abraham, 1998) to create baseline models reflecting natural element concentrations. This separation is achieved through normalization, often using conservative elements to normalize trace metal geochemical data (Sutherland, 2000). Quantitative techniques such as the geoaccumulation index (I_geo_) and contamination factor (CF) are examples of these methods.

The I_geo_, developed by Muller (1969) to evaluate metal pollution in sediments by comparing current concentrations with pre-industrial levels, has been widely used in studies on environmental contamination (Meng et al., 2020; Niu et al., 2021). An ecological risk index, which quantifies the ecological risk of specific pollutants in an environment, can be created by analyzing heavy metal levels (Hakanson, 1980; Sharma et al., 2007). Due to the absence of pre-industrial values in this study, continental crust values were used for Igeo calculations (Mudimbu et al., 2022).

Table 7 presents the GCIs calculated in this study along with the parameters used. In Table 7, C_n_ represents the sample concentration of heavy metals, C_ref_ is the background concentration level, and B_n_ is the mean concentration level in the Earth's crust, which includes 98 for V, 126 for Cr, 716 for Mn, 43200 for Fe, 24 for Co, 56 for Ni, 25 for Cu, 65 for Zn, 1.7 for As, 333 for Sr, 1.1 for Mo, 0.1 for Cd, 23 for Sn, 0.3 for Sb, 584 for Ba, 0.52 for Tl, and 14.8 for Pb (Wedepohl, 1995). B_ref_ is the background concentration level for the reference metal (Matthai & Birch, 2001) and T_i_ is the toxic-response factor for the metal. The following Ti values are provided by Håkanson (Hakanson, 1980) and Xu et al. (Xu et al., 2008): V-2, Cr-2, Co-5, Ni-5, Mn-1, Tl-10, Cu-5, Zn-1, As-10, Cd-30, and Pb-5.

**Table 7:** Geochemical indices used in this study

Index	Formula	Risk categories
Geoaccumulation index	$\text{Igeo}={log}_{2} ({C}_{n}/1.5{C}_{ref})$	1. Unpolluted (i_geo_ < 0)
		2. Unpolluted to moderately polluted (i_geo_ = 0–1)
		3. Moderately polluted (i_geo_ = 1–2)
		4. Moderately to strongly polluted (i_geo_ = 2–3)
		5. Highly polluted (i_geo_ = 3–4)
		6. Highly to extremely polluted (i_geo_ = 4–5)
		7. Extremely polluted (i_geo_ > 5)
Enrichment factor	$EF=\frac{{C}_{n}/{C}_{ref}}{{B}_{n}/{B}_{ref}}$	1. Minimal (ef < 2)
		2. Moderate (ef = 2–5)
		3. Significant (ef = 5–20)
		4. Very high (ef = 20–40)
		5. Extreme (ef > 40)
Ecological risk factor	${Er}_{i}= {T}_{i} \times \frac{{C}_{n}}{{C}_{ref}}$	1. Low (er_i_ < 40)
		2. Moderate (er_i_ = 40–80)
		3. Considerable (er_i_ = 80–160)
		4. High (er_i_ = 160–320)
		5. Very high (er_i_ > 320)
Potential ecological risk	$RI=\sum {Er}_{i}$	1. Low (ri < 150)
		2. Moderate (ri = 150–300)
		3. Considerable (ri = 300– 600)
		4. High (ri > 600)

### Statistical analysis

HM content underwent multivariate statistical analysis, encompassing Pearson's correlation matrix and factor analysis. This approach enables the identification of any correlations between different HMs present in the surface dust samples examined in this study. Such interactions provide crucial insights into the behavior of HMs and pathways within the system. The statistical analyses were conducted utilizing IBM SPSS (Version 26.0) statistical software.

## Results and discussion

### Heavy metal concentrations of the surface dust samples

The concentrations of sixteen HMs in surface dust around the cement factory are summarized in Table S1 (refer to SI), which presents statistics including minimum, maximum, mean, standard deviation, and relative variables.

**Fig. 2:**
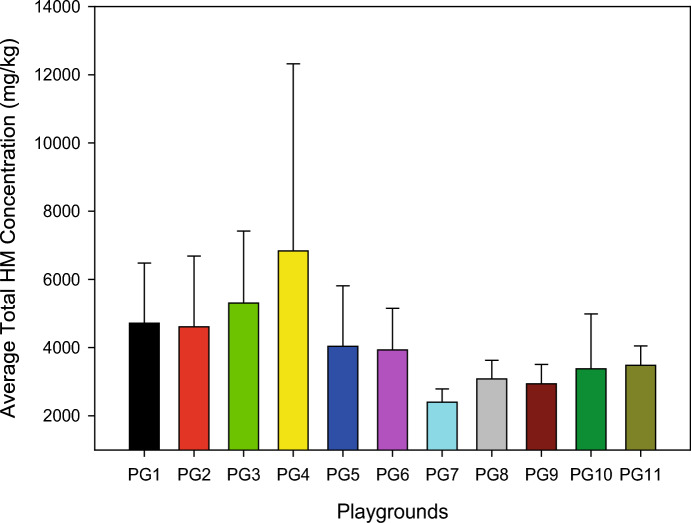
Average total HM concentration values of the sampling sites

The predominant metals in the dust samples were found to be Fe and Zn, with average concentrations exceeding 1650 mg/kg. Concentration trends indicated lower loads of metals in the order of Tl < Cd < Sb < Co < Sn < Pb < As, all below 5 mg/kg, except Be and Se. Figure 2 illustrates the average total heavy metal concentrations at various sampling sites.

The highest average HM concentrations were observed at PG4, PG3, PG2, and PG1, located within the ExIA. Sites close to ExIA, such as PG5 and PG6, showed relatively similar results, whereas PG7, PG9, and PG11, situated outside the EIA boundaries, exhibited lower concentrations. PG8 and PG10, within EIA boundaries, displayed concentrations similar to each other, with PG7 showing the lowest levels. The highest concentration was recorded at PG4 (6832 mg/kg), and the lowest at PG7 (2401 mg/kg).

Cr concentrations in the range of 2.07 to 232.46 mg/kg, with an average of 23.47 mg/kg, were observed. The primary origins of Cr in cement stem from natural raw materials utilized in clinker production, with expected Cr concentrations in clinker falling within the 20–30 ppm range. Additionally, Cr is sourced from hard coal, a fuel used in the process, which contains an average Cr concentration of 25 ppm (Gragarczyk & Wardak, 2022). The presence of Cr in surface dust samples analyzed is likely due to its release through friction, wear, and tear from the linings of cement industry rotary equipment (Banat et al., 2005). Variations in Cr levels across the examined samples, illustrated in Figure S1 of the SI, reveal the highest and lowest concentrations in PG4 and PG7, respectively.

Pb concentrations ranged from 0.07 to 19.07 mg/kg, with an average of 2.05 mg/kg. While some metals become trapped within the clinker, others are volatilized and then condensed onto dust particles (Schuhmacher et al., 2009). Cement factory emissions have been found to contain Cd, Cr, Cu, Mn, Pb, and Zn (Arfala et al., 2018; Bermudez et al., 2010), which can potentially be dispersed by wind and significantly impact the ecosystem. Analyzing the fluctuations in average Pb concentrations across sampling sites, the highest levels were observed in PG4 and PG1, while the lowest value was recorded in PG8 (Figure S1). Arslan (2001) investigated HM levels in Bursa (in street dust), noting average Pb concentrations ranging from 35 to 485 mg/kg at various sampling locations, with relatively lower values reported in this study.

Cu concentrations ranged from 0.74 to 51.71 mg/kg, with an average of 11.37 mg/kg. The levels of Cu detected in surface dust samples could have been influenced by emissions from the cement plant and exhaust from trucks and vehicles engaged in various activities near the sampling sites (Odoh et al., 2014). Another potential source of Cu in the dust samples is mechanical wear and tear from vehicles (Al-Khashman, 2007). The average Cu concentration values across sampling sites, listed from highest to lowest, are as follows: PG4 > PG1 > PG3 > PG5 > PG6 > PG2 > PG11 > PG7 > PG10 > PG9 > PG8, as depicted in Figure S1.

The mean concentration of Zn in the surface dust samples examined was 2298.28 mg/kg. Carreras and Pignata (2002) and Al-Khashman (2007) have linked the amount of Zn in dust to a variety of issues, including vehicle emissions and tire wear and tear. A study on road dust collected from Bursa in 2001 found Zn levels ranging from 11 to 121 mg/kg. Other potential sources of Zn accumulation in dust include corrosion of galvanized playground equipment, lubricants, and atmospheric deposition resulting from coal-burning processes (Arslan, 2001).

Ni concentrations in the surface dust samples analyzed ranged from 7.97 to 81.64 mg/kg, with an average concentration of 16.58 mg/kg. The accumulation of Ni in the studied surface dust may be attributed to cement manufacturing and other human-related activities occurring in the vicinity of playgrounds. Industrial emissions and agricultural practices are additional contributors to Ni buildup in surface dust. Elevated exposure to Ni in dust can pose health risks, including lung fibrosis, skin allergies, and cancer. Therefore, careful attention should be paid to Ni concentrations in dust samples surrounding cement factories (Cempel and Nickel 2006).

Mn was found to be the most prevalent element in the surface dust samples examined, with an average concentration of 30.54 mg/kg. Mn naturally occurs in relatively high concentrations of dust under normal conditions (Idris, 2008). Hence, the presence of Mn in the analyzed dust cannot be solely attributed to human activities, nor can manganese be classified as a pollutant. The spatial distribution of these metals underscores the influence of cement manufacturing activities, coal combustion processes, and vehicular emissions near playgrounds. Detailed concentration levels of HM species at each sampling site are provided in Table S1.

Figure S1 depicts variations in concentration by heavy metal species. The highest average concentration value was obtained for Fe, and Cd is the species with the lowest average concentration value. Data on species detected in dust samples collected from each playground are shown in Figure S2. It was determined that Fe and Zn were dominant on a species basis.

Comparison of the levels of Cr, Cu, Zn, Pb, Cd, As, and Ni in surface dust samples to the recommended limits (Table 7) of the Ministry of Environment and Urbanization of Türkiye Regulation on Soil Pollution Control and Point Source Contaminated Areas (MoEU, 2010) showed that the concentration of As in the surface dust samples was beyond the accepted limit.

While concentrations of pollutants fluctuate across different sites, it was noted that pollutant levels decrease with increasing distance from the cement factory. Similar observations have been documented in other studies reported in the literature (Mandal & Voutchkov, 2011). There are studies in the literature where heavy metal concentrations are determined in the soil around cement factories or bioindicator organisms (Bermudez et al., 2010; Cutillas-Barreiro et al., 2016; Elik & Akçay, 2000; Saltalı et al., 2018; Ünal et al., 2011; Wang et al., 2018a, 2018b, 2018c). Semhi et al. (2010) stated that the cement factory affected the heavy metal content of plant and soil samples in the 2 km area around it. It is clear that cement factories play a dominant role in metal pollution, and determined values vary depending on the distance to the source (Table 8).

**Table 8:** List of generic pollutant limit values, (MoEU, 2010)

Pollutant	CAS No	Absorption through ingestion of soil and skin contact (mg/kg oven-dry soil)	Outdoor Inhalation of volatile substances (mg/kg oven-dry soil)	Inhalation of fugitive dust outdoors (mg/kg oven-dry soil)
As	7440–38-2	0,4^b,c^	–	471^e^
Cu	7440–50-8	3129^b^^,c^	–	–^f^
Zn	7440–66-6	23464^b^^,c^	–	–^f^
Cd	7440–43-9	70^b^^,m^	–	1124^e^
Co	7440–48-4	23^b^^,c^	–	225^e^
Cr (III)	16,065–83-1	117321^b^^,c^	–	–^f^
Cr (VI)	18,540–29-9	235^b^^,c^	–	24^e^
Cr (Total)^g^	7440–47-3	235^b^^,c^	–	24^e^
Pb	7439–92-1	400^n^	–	–^f^
Ni	7440–02-0	1564^b^^,c^	–	–^f^

^b^In calculating this value, the hazard index is accepted as “1”

^c^Since there is no skin absorption factor for this pollutant, only the soil ingestion exposure route was taken into account

^e^In calculating this value, the cancer risk was accepted as "10^–6^"

^f^o toxicological value is available for this route of exposure

^m^In calculating this limit value, the value determined for Cadmium to be taken into the body through food was used

^n^his value is taken from US EPA, 1994 (US EPA, 1994. Revised Interim Soil Lead Guidance for CERCLA Sites and RCRA Corrective Action Facilities, EPA/540/F-94/043, Office of Solid Waste and Emergency Response, Washington, D.C. Directive 9355.4–12.)

^g^Limit values calculated for chromium (VI) were used

The levels of HM contamination in surface dust and soil that children may encounter in playgrounds in Türkiye and other regions are shown in Table S2. Factors such as city size, age, population, traffic density, and industrial activity can impact the concentrations and distribution of HMs (Donado et al., 2021; Rodríguez-Oroz et al., 2018).

Previous studies have reported concentrations of V, Cr, Co, Ni, Cu, Zn, As, Sr, Cd, Sb, Tl, and Pb in soil samples ranging from 9.50–60.9 mg/kg, 3.09–201 mg/kg, 1.87–16.9 mg/kg, 4.43–57.2 mg/kg, 4.89–43.8 mg/kg, 20.9–237 mg/kg, 0.110–18.9 mg/kg, 43.0–49.0 mg/kg, 0.038–4.5 mg/kg, 0.600–0.950 mg/kg, 0.100–0.200 mg/kg, and 1.94–195 mg/kg, respectively. For dust samples, Cr, Ni, Cu, Zn, Cd, and Pb concentrations ranged from 16–263 mg/kg, 6.8–13 mg/kg, 0.600–201 mg/kg, 11.2–1883 mg/kg, 2.00–7.00 mg/kg, and 8.00–302 mg/kg, respectively (Table S2). The concentration values detected in surface dust samples in this study were consistent with those from earlier research.

The levels of Cr, Ni, Cu, and Pb in dust samples from Bursa, Türkiye, were significantly lower than those found in large megacities like Istanbul (Türkiye) (Ak et al., 2012), Madrid (Spain) (De Miguel et al., 2007), and Hong Kong (China) (Chen et al., 1997, Ng et al. 2003), which are heavily affected by traffic and atmospheric deposition, the primary sources of these metals. However, higher heavy metal levels were observed in Bursa compared to Canakkale (Parlak et al., 2022) and Riyadh (Alotaibi et al., 2006) (Table S2).

The results of the statistical analysis carried out to determine whether there is a difference between the HM concentrations detected in the dust samples and to determine the source of the existing pollution are explained below. The normality tests conducted on the collected data revealed that the data did not follow a normal distribution. The kurtosis and skewness values of HMs are not within the range of ± 1.5 (Tabachnick 2013). Similarly, it is stated in the literature that the kurtosis and skewness values divided by the standard error should be between ± 1.96 and 1.96. In addition, for the data to show a normal distribution, the *p* values in the Kolmogorov–Smirnov test must be higher than 0.05 (*p* > 0.05) (Büyüköztürk, 2011). The *p* values of HMs obtained with the Kolmogorov–Smirnov test are less than 0.05 (*p* < 0.05) in this study.

Spearman correlation analysis (SCA) serves as a statistical method utilized when variables exhibit a non-normal distribution, aiming to establish correlations between elements (Liang et al., 2017). Elements with a *p* value less than 0.05 signify a notable correlation between them. The outcomes of the SCA for HMs are outlined in Table 9. Within the SCA results, metals demonstrating a positive relationship are highlighted in bold in the provided table. A significant positive correlation was observed among Cr, Mn, Fe, Co, Cu, Pb, As, and Ni (*p* < 0.01), except for Zn and Cd. Additionally, Cu and Pb exhibited a correlation (*p* < 0.05), suggesting a potential shared source of contamination, such as anthropogenic activities, metallic waste corrosion, vehicular emissions, and pollution from cement production processes. Conversely, Zn and Cd displayed a weak correlation with all studied metals, indicating their likely derivation from natural sources (Idris, 2008).

**Table 9:** Spearman correlation coefficient analysis of heavy metal contents in surface dust samples

Correlations^c^												
			Cr	Mn	Fe	Co	Cu	Zn	Pb	Cd	As	Ni
Spearman's rho	Cr	Correlation Coefficient	1.000	.769^**^	.741^**^	.524^**^	.445^**^	0.203	.490^**^	0.041	.687^**^	.635^**^
		Sig. (2 − tailed)		0.000	0.000	0.001	0.008	0.249	0.003	0.820	0.000	0.000
	Mn	Correlation Coefficient	.769^**^	1.000	.864^**^	.724^**^	.520^**^	0.282	0.273	− 0.077	.845^**^	.743^**^
		Sig. (2 − tailed)	0.000		0.000	0.000	0.002	0.107	0.119	0.667	0.000	0.000
	Fe	Correlation Coefficient	.741^**^	.864^**^	1.000	.581^**^	.479^**^	0.172	0.206	− 0.051	.712^**^	.597^**^
		Sig. (2 − tailed)	0.000	0.000		0.000	0.004	0.332	0.242	0.773	0.000	0.000
	Co	Correlation Coefficient	.524^**^	.724^**^	.581^**^	1.000	0.269	0.248	0.203	0.210	.664^**^	.549^**^
		Sig. (2 − tailed)	0.001	0.000	0.000		0.124	0.158	0.248	0.233	0.000	0.001
	Cu	Correlation Coefficient	.445^**^	.520^**^	.479^**^	0.269	1.000	0.217	.404^*^	− 0.111	.711^**^	.530^**^
		Sig. (2 − tailed)	0.008	0.002	0.004	0.124		0.217	0.018	0.531	0.000	0.001
	Zn	Correlation Coefficient	0.203	0.282	0.172	0.248	0.217	1.000	0.080	− 0.180	0.299	0.286
		Sig. (2 − tailed)	0.249	0.107	0.332	0.158	0.217		0.651	0.309	0.086	0.101
	Pb	Correlation Coefficient	.490^**^	0.273	0.206	0.203	.404^*^	0.080	1.000	0.175	.455^**^	0.239
		Sig. (2 − tailed)	0.003	0.119	0.242	0.248	0.018	0.651		0.322	0.007	0.173
	Cd	Correlation Coefficient	0.041	− 0.077	− 0.051	0.210	− 0.111	− 0.180	0.175	1.000	− 0.030	0.118
		Sig. (2 − tailed)	0.820	0.667	0.773	0.233	0.531	0.309	0.322		0.867	0.505
	As	Correlation Coefficient	.687^**^	.845^**^	.712^**^	.664^**^	.711^**^	0.299	.455^**^	− 0.030	1.000	.760^**^
		Sig. (2 − tailed)	0.000	0.000	0.000	0.000	0.000	0.086	0.007	0.867		0.000
	Ni	Correlation Coefficient	.635^**^	.743^**^	.597^**^	.549^**^	.530^**^	0.286	0.239	0.118	.760^**^	1.000
		Sig. (2 − tailed)	0.000	0.000	0.000	0.001	0.001	0.101	0.173	0.505	0.000	

**Correlation is significant (written in bold) at the 0.01 level (2 − tailed). *Correlation is significant at the 0.05 level (2 − tailed). c. Listwise N = 34

**Fig. 3:**
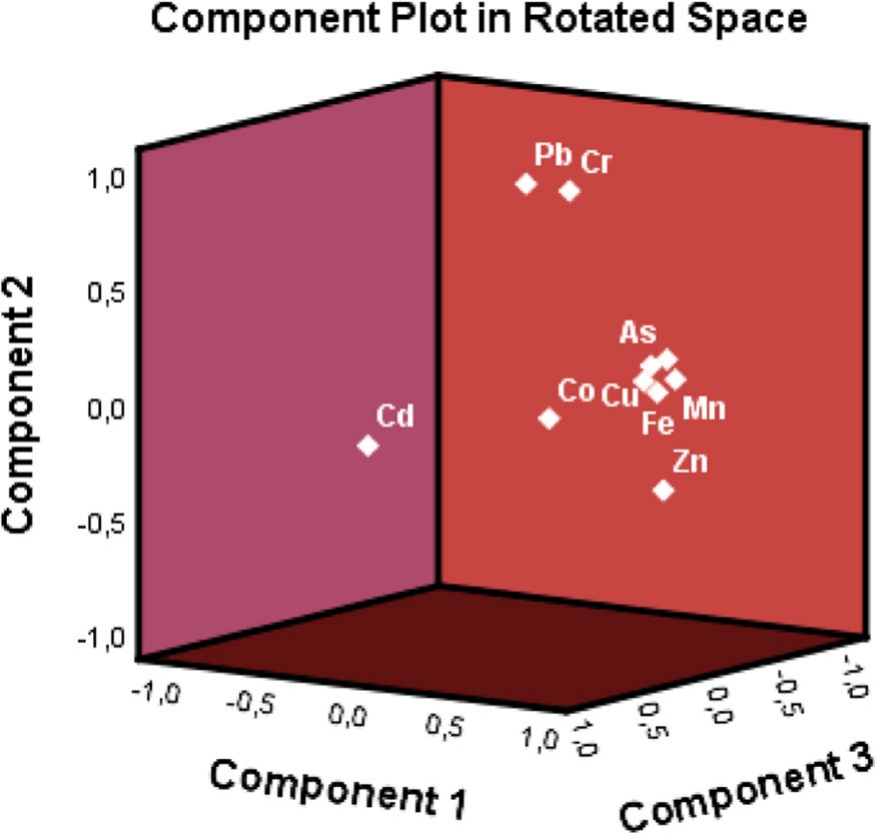
The scree plot of the PCA

Principal component analysis (PCA) stands as a widely utilized multivariable statistical technique aimed at enhancing the clarity and precision of insights into the origin and dispersion of HMs in surface dust (Roy et al., 2019; Shabbaj et al., 2018). The correlation matrix collected underwent variance rotation with Kaiser normalization. The results of factor loadings obtained through Varimax rotation, along with their respective percentages of variance and commonalities, are presented in Table 10. Both the Kaiser–Meyer–Olkin (KMO) value (0.687) and Bartlett’s test (*p* < 0.001) confirmed the validity and appropriateness of the PCA results. The PCA findings revealed three primary components with eigenvalues exceeding 1. These three principal components (PCs) collectively explained over 79.905% of the data variance. Figure 3 illustrates the scree plot of the PCA.

**Table 10:** The result of principal component analysis

Rotated component matrix^a^			
	Component		
	PC1	PC2	PC3
Mn	0.951		
As	0.904		
Ni	0.874		
Fe	0.793		
Cu	0.739		
Co	0.670		0.610
Zn	0.644	− 0.397	
Pb		0.920	
Cr		0.899	
Cd			0.938

Extraction Method: Principal Component Analysis

Rotation Method: Varimax with Kaiser Normalization

^a^Rotation converged in 5 iterations

The first component, PC1, was predominantly influenced by Mn, As, Ni, Fe, Cu, Co, and Zn, accounting for 49.276% of the total variance. This component highlighted the impact of human activities such as coal combustion, limestone processing near the factory, tire, brake wear, and diesel exhaust emissions as the primary sources of these metals (Dytłow & Górka-Kostrubiec, 2021; Hini et al., 2019; Kabadayi & Cesur, 2010; Kormoker et al., 2021).

Component PC2 exhibited a significant loading of Pb and Cr, contributing 17.570% to the overall variance. This observation aligns with the positive correlation between Pb and Cr. Additionally, Hini et al. (2019) suggested that Cr might originate from vehicle tires due to friction with the road surface. Consequently, PC2 was identified as indicative of vehicle emissions. The third component, PC3, was primarily characterized by Cd, which accounted for 13.059% of the overall variance. Possible origins of Cd in surface dust may include diesel fuel, brake pads, and car paints (Wahab et al., 2020).

### Non-carcinogenic health risk assessment

Hazard quotients (HQ) and Hazard Index (HI) estimates for children, assessing non-carcinogenic risks through three exposure routes, were computed using Eqs. (1)–(5). The outcomes are showcased in Table 11. Notably, all evaluated heavy metals (HMs) exhibited HQ values below the safe threshold (HQ < 1), and the non-carcinogenic health risk values of each HM in surface dust samples were deemed acceptable.

**Table 11:** Non-carcinogenic (three exposure routes) risk for children

	Cr	Mn	Fe	Co	Cu	Zn	Pb	Cd	As	Ni
HQ_inh_										
PG1	7.8 X10^−4^	4.2 X10^−4^	1.7 X10^−2^	1.2 X10^−4^	5.3 X10^−6^	4.3 X10^−6^	8.8 X10^−7^	1.60 X10^−7^	9.39 X10^−6^	5.85 X10^−7^
PG2	4.7 X10^−4^	3.4 X10^−4^	2.2 X10^−2^	1.2 X10^−4^	3.7 X10^−6^	3.3 X10^−6^	1.5 X10^−7^	1.36 X10^−7^	6.65 X10^−6^	4.78 X10^−7^
PG3	4.1 X10^−4^	4.2 X10^−4^	2.6 X10^−2^	1.1 X10^−4^	4.7 X10^−6^	3.8 X10^−6^	1.0 X10^−7^	5.61 X10^−8^	8.58 X10^−6^	4.20 X10^−7^
PG4	1.4 X10^−3^	6.2 X10^−4^	3.8 X10^−2^	1.8 X10^−4^	8.3 X10^−6^	3.9 X10^−6^	9.9 X10^−7^	1.76 X10^−7^	1.36 X10^−5^	6.72 X10^−7^
PG5	2.0 X10^−4^	1.5 X10^−4^	8.9 X10^−3^	4.7 X10^−5^	3.8 X10^−6^	4.7 X10^−6^	1.9 X10^−7^	7.22 X10^−8^	2.98 X10^−6^	2.68 X10^−7^
PG6	1.7 X10^−4^	1.9 X10^−4^	1.1 X10^−2^	4.3 X10^−5^	3.7 X10^−6^	4.2 X10^−6^	1.1 X10^−7^	1.20 X10^−7^	3.68 X10^−6^	2.37 X10^−7^
PG7	9.5 X10^−5^	9.6 E-05	5.4 X10^−3^	1.4 X10^−5^	2.7 X10^−6^	2.8 X10^−6^	1.8 X10^−7^	1.76 X10^−7^	1.12 X10^−6^	2.51 X10^−7^
PG8	1.9 X10^−4^	2.2 X10^−4^	1.2 X10^−2^	8.2 X10^−5^	1.6 X10^−6^	2.8 X10^−6^	4.1 X10^−8^	3.21 X10^−7^	2.49 X10^−6^	2.61 X10^−7^
PG9	1.7 X10^−4^	3.3 X10^−4^	1.2 X10^−2^	1.1 X10^−4^	2.3 X10^−6^	2.4 X10^−6^	1.1 X10^−7^	3.34 X10^−7^	2.85 X10^−6^	2.47 X10^−7^
PG10	1.3 X10^−4^	1.0 X10^−4^	5.4 X10^−3^	2.8 X10^−5^	2.5 X10^−6^	4.3 X10^−6^	1.1 X10^−7^	1.80 X10^−7^	9.91 X10^−6^	4.56 X10^−7^
PG11	2.1 X10^−4^	2.8 X10^−4^	1.2 X10^−2^	1.1 X10^−4^	3.4 X10^−6^	3.2 X10^−6^	1.6 X10^−7^	1.44 X10^−7^	6.62 X10^−6^	2.86 X10^−7^
HQ_ing_										
PG1	2.0 × 10^−1^	4.1 × 10^−3^	3.3 × 10^−2^	6.4 × 10^−2^	5.0 × 10^−3^	1.2 × 10^−1^	2.4 × 10^−2^	4.36 × 10^−3^	2.55 × 10^−1^	1.64 × 10^−2^
PG2	1.2 × 10^−1^	3.3 × 10^−3^	4.3 × 10^−2^	6.6 × 10^−2^	3.6 × 10^−3^	9.0 × 10^−2^	4.2 × 10^−3^	3.71 × 10^−3^	1.81 × 10^−1^	1.34 × 10^−2^
PG3	1.1 × 10^−1^	4.1 × 10^−3^	5.1 × 10^−2^	6.0 × 10^−2^	4.4 × 10^−3^	1.0 × 10^−1^	2.8 × 10^−3^	1.53 × 10^−3^	2.33 × 10^−1^	1.18 × 10^−2^
PG4	3.7 × 10^−1^	6.0 × 10^−3^	7.5 × 10^−2^	9.8 × 10^−2^	7.9 × 10^−3^	1.1 × 10^−1^	2.7 × 10^−2^	4.80 × 10^−3^	3.70 × 10^−1^	1.88 × 10^−2^
PG5	5.2 × 10^−2^	1.5 × 10^−3^	1.7 × 10^−2^	2.6 × 10^−2^	3.6 × 10^−3^	1.3 × 10^−1^	5.1 × 10^−3^	1.96 × 10^−3^	8. × 10 × 10^−2^	7.50 × 10^−3^
PG6	4.5 × 10^−2^	1.8 × 10^−3^	2.1 × 10^−2^	2.3 × 10^−2^	3.5 × 10^−3^	1.1 × 10^−1^	3.0 × 10^−3^	3.27 × 10^−3^	1.00 × 10^−1^	6.65 × 10^−3^
PG7	2.5 × 10^−2^	9.4 × 10^−4^	1.1 × 10^−2^	7.3 × 10^−3^	2.6 × 10^−3^	7.6 × 10^−2^	5.0 × 10^−3^	4.80 × 10^−3^	3.03 × 10^−2^	7.03 × 10^−3^
PG8	4.9 × 10^−2^	2.1 × 10^−3^	2.3 × 10^−2^	4.4 × 10^−2^	1.6 × 10^−3^	7.5 × 10^−2^	1.1 × 10^−3^	8.73 × 10^−3^	6.77 × 10^−2^	7.31 × 10^−3^
PG9	4.3 × 10^−2^	3.2 × 10^−3^	2.4 × 10^−2^	6.2 × 10^−2^	2.2 × 10^−3^	6.5 × 10^−2^	3.0 × 10^−3^	9.09 × 10^−3^	7.76 × 10^−2^	6.91 × 10^−3^
PG10	3.4 × 10^−2^	9.7 × 10^−4^	1.1 × 10^−2^	1.5 × 10^−2^	2.4 × 10^−3^	1.2 × 10^−1^	2.9 × 10^−3^	4.91 × 10^−3^	2.70 × 10^−1^	1.28 × 10^−2^
PG11	5.3 × 10^−2^	2.7 × 10^−3^	2.4 × 10^−2^	5.7 × 10^−2^	3.3 × 10^−3^	8.8 × 10^−2^	4.3 × 10^−3^	3.93 × 10^−3^	1.80 × 10^−1^	8.03 × 10^−3^
HQ_der_										
PG1	1.9 X10^−2^	6.5 X10^−6^	5.2 X10^−5^	1.0 X10^−4^	2.7 X10^−6^	9.4 X10^−4^	2.6 X10^−4^	6.98 X10^−4^	4.09 X10^−4^	9.71 X10^−5^
PG2	1.2 X10^−2^	5.3 X10^−6^	6.9 X10^−5^	1.0 X10^−4^	1.9 X10^−6^	7.2 X10^−4^	4.5 X10^−5^	5.93 X10^−4^	2.90 X10^−4^	7.94 X10^−5^
PG3	1.0 X10^−2^	6.5 X10^−6^	8.1 X10^−5^	9.6 X10^−5^	2.4 X10^−6^	8.2 X10^−4^	2.9 X10^−5^	2.44 X10^−4^	3.73 X10^−4^	6.97 X10^−5^
PG4	3.5 X10^−2^	9.6 X10^−6^	1.2 X10^−4^	1.6 X10^−4^	4.2 X10^−6^	8.5 X10^−4^	2.9 X10^−4^	7.68 X10^−4^	5.92 X10^−4^	1.12 X10^−4^
PG5	5.0 X10^−3^	2.3 X10^−6^	2.8 X10^−5^	4.1 X10^−5^	1.9 X10^−6^	1.0 X10^−3^	5.4 X10^−5^	3.14 X10^−4^	1.30 X10^−4^	4.45 X10^−5^
PG6	4.3 X10^−3^	2.9 X10^−6^	3.4 X10^−5^	3.7 X10^−5^	1.9 X10^−6^	9.1 X10^−4^	3.2 X10^−5^	5.23 X10^−4^	1.60 X10^−4^	3.94 X10^−5^
PG7	2.4 X10^−3^	1.5 X10^−6^	1.7 X10^−5^	1.2 X10^−5^	1.4 X10^−6^	6.1 X10^−4^	5.3 X10^−5^	7.68 X10^−4^	4.85 X10^−5^	4.16 X10^−5^
PG8	4.7 X10^−3^	3.4 X10^−6^	3.6 X10^−5^	7.1 X10^−5^	8.3 X10^−7^	6.0 X10^−4^	1.2 X10^−5^	1.40 X10^−3^	1.08 X10^−4^	4.33 X10^−5^
PG9	4.1 X10^−3^	5.2 X10^−6^	3.9 X10^−5^	1.0 X10^−4^	1.2 X10^−6^	5.2 X10^−4^	3.2 X10^−5^	1.45 X10^−3^	1.24 X10^−4^	4.10 X10^−5^
PG10	3.3 X10^−3^	1.6 X10^−6^	1.7 X10^−5^	2.5 X10^−5^	1.3 X10^−6^	9.3 X10^−4^	3.1 X10^−5^	7.85 X10^−4^	4.31 X10^−4^	7.57 X10^−5^
PG11	5.1 X10^−3^	4.3 X10^−6^	3.9 X10^−5^	9.2 X10^−5^	1.7 X10^−6^	7.0 X10^−4^	4.6 X10^−5^	6.28 X10^−4^	2.88 X10^−4^	4.76 X10^−5^
HI										
PG1	2.2 X10^−1^	4.5 X10^−3^	4.9 X10^−2^	6.4 X10^−2^	5.1 X10^−3^	1.2 X10^−1^	2.4 X10^−2^	5.06 X10^−3^	2.56 X10^−1^	1.65 X10^−2^
PG2	1.3 X10^−1^	3.6 X10^−3^	6.5 X10^−2^	6.6 X10^−2^	3.6 X10^−3^	9.1 X10^−2^	4.2 X10^−3^	4.30 X10^−3^	1.81 X10^−1^	1.35 X10^−2^
PG3	1.2 X10^−1^	4.5 X10^−3^	7.7 X10^−2^	6.0 X10^−2^	4.5 X10^−3^	1.0 X10^−1^	2.8 X10^−3^	1.77 X10^−3^	2.34 X10-^1^	1.18 X10^−2^
PG4	4.1 X10^−1^	6.6 X10^−3^	1.1 X10^−1^	9.8 X10^−2^	7.9 X10^−3^	1.1 X10^−1^	2.7 X10^−2^	5.57 X10^−3^	3.70 X10^−1^	1.89 X10^−2^
PG5	5.7 X10^−2^	1.6 X10^−3^	2.6 X10^−2^	2.6 X10^−2^	3.6 X10^−3^	1.3 X10^−1^	5.1 X10^−3^	2.28 X10^−3^	8.11 X10^−2^	7.55 X10^−3^
PG6	4.9 X10^−2^	2.0 X10^−3^	3.2 X10^−2^	2.3 X10^−2^	3.5 X10^−3^	1.2 X10^−1^	3.1 X10^−3^	3.80 X10^−3^	1.00 X10^−1^	6.69 X10^−3^
PG7	2.7 X10^−2^	1.0 X10^−3^	1.6 X10^−2^	7.4 X10^−3^	2.6 X10^−3^	7.6 X10^−2^	5.1 X10^−3^	5.57 X10^−3^	3.04 X10^−2^	7.07 X10^−3^
PG8	5.4 X10^−2^	2.3 X10^−3^	3.5 X10^−2^	4.5 X10^−2^	1.6 X10^−3^	7.5 X10^−2^	1.1 X10^−3^	1.01 X10^−2^	6.78 X10^−2^	7.35 X10^−3^
PG9	4.7 X10^−2^	3.6 X10^−3^	3.7 X10^−2^	6.2 X10^−2^	2.2 X10^−3^	6.6 X10^−2^	3.1 X10^−3^	1.05 X10^−2^	7.77 X10^−2^	6.95 X10^−3^
PG10	3.8 X10^−2^	1.1 X10^−3^	1.6 X10^−2^	1.5 X10^−2^	2.4 X10^−3^	1.2 X10^−1^	2.9 X10^−3^	5.69 X10^−3^	2.70 X10^−1^	1.29 X10^−2^
PG11	5.9 X10^−2^	3.0 X10^−3^	3.7 X10^−2^	5.8 X10^−2^	3.3 X10^−3^	8.8 X10^−2^	4.3 X10^−3^	4.55 X10^−3^	1.80 X10^−1^	8.07 X10^−3^

The highest HQ_inh_ value found was 3.8 × 10^–2^ for Fe, whereas the lowest was 4.1 × 10^–8^ for Pb. For HQ_ing_ and HQ_dermal_ parameters, the maximum levels identified were 4.1 × 10^–8^ for As and 3.5 × 10^–2^ for Cr, with the lowest levels being 9.4 × 10^–4^ for Mn and 8.3 × 10^–7^ for Cu, respectively. Furthermore, the HQ values across the three exposure pathways generally followed an order of ingestion > dermal contact > inhalation, except for Fe, Co, and Cu, which exhibited an order of ingestion > inhalation > dermal contact for children. The HI values for the assessed HMs ranged from 1 × 10^–3^ to 4.1 × 10^–1^, with an average of 5 × 10^–2^. Additionally, the HI values for individual HMs among children were consistently lower than unity (HI < 1), signifying that the non-carcinogenic risks posed by these metals in surface dust samples collected around cement factories are negligible. Upon analyzing cumulative hazard index values across the sampling sites for metal exposure through the three routes, the highest and lowest values were observed in the PG4 (1.16 × 10^0^) and PG7 (1.78 × 10^–1^) regions, respectively, suggesting a lower potential non-carcinogenic risk associated with heavy metal exposure for children.

According to the classification system by the International Agency for Research on Cancer (IARC), as cited in Cao et al. (2016), Zn, Pb, Cr, Cd, Cu, and Mn are categorized as non-carcinogenic pollutants, whereas Ni, As, Cr, Cd, and Co are considered potentially carcinogenic based on research by Izquierdo et al. (2015). This study specifically assessed the cancer risk associated with Ni, As, Cr, and Cd. Table 12 presents the Incremental Lifetime Cancer Risk (ILCR) posed by each contaminant observed in surface dust samples to local children. The lowest and highest ILCR values were identified as 3.42 × 10^–10^ for Cd and 1.71 × 10^–6^ for Cr, respectively, with a mean ILCR value of 1.18 × 10^–7^ across the collected samples. The cumulative ILCR values ranged from 1.2 × 10^–7^ (at PG7) to 1.73 × 10^–6^ (at PG4), with a mean value of 4.74 × 10^–7^. However, the carcinogenic risk levels for children generally fell within the acceptable range for carcinogens, which typically ranges from 10^–6^ to 10^–4^ according to Li et al. (2017). None of the dust samples analyzed for potential carcinogenicity exceeded tolerable levels.

**Table 12:** ILCR values of carcinogen HMs

ILCR	Cr	Cd	As	Ni
PG1	9.35 × 10^−7^	9.78 × 10^−10^	4.23 × 10^−9^	1.01 × 10^−8^
PG2	5.62 × 10^−7^	8.32 × 10^−9^	2.99 × 10^−9^	8.28 × 10^−9^
PG3	4.92 × 10^−7^	3.42 × 10^−10^	3.86 × 10^−9^	7.27 × 10^−9^
PG4	1.71 × 10^−6^	1.08 × 10^−9^	6.12 × 10^−9^	1.16 × 10^−8^
PG5	2.42 × 10^−7^	4.40 × 10^−10^	1.34 × 10^−9^	4.63 × 10^−9^
PG6	2.08 × 10^−7^	7.34 × 10^−10^	1.66 × 10^−9^	4.11 × 10^−9^
PG7	1.14 × 10^−7^	1.08 × 10^−9^	5.02 × 10^−10^	4.34 × 10^−9^
PG8	2.26 × 10^−7^	1.96 × 10^−9^	1.12 × 10^−9^	4.51 × 10^−9^
PG9	2.00 × 10^−7^	2.04 × 10^−9^	1.28 × 10^−9^	4.27 × 10^−9^
PG10	1.59 × 10^−7^	1.10 × 10^−9^	4.46 × 10^−9^	7.89 × 10^−9^
PG11	2.48 × 10^−7^	8.81 × 10^−10^	2.98 × 10^−9^	4.96 × 10^−9^

It's important to recognize that carcinogenic pollutants found in urban surface dust extend beyond the HMs examined in this study. Despite findings indicating a generally low carcinogenic risk associated with HMs in playground surface dust in Bursa City and no evident risk for children, it's worth noting that various other contaminants, such as arsenic (As) and polycyclic aromatic hydrocarbons (PAHs), can also contribute to cancer risks (Wang et al., 2018a, 2018b, 2018c).

Therefore, the study's assessment of the carcinogenic risk posed by HMs in playground dust in Bursa City might not fully capture the overall picture. Additionally, the types of HMs present and their ecological toxicity play a significant role beyond just their concentration. Subsequent research efforts will need to consider these factors to provide a more precise evaluation of the health risks associated with these metals.

### Geoaccumulation index (I_geo_)

In the surface dust samples, the geoaccumulation index (I_geo_) indicated an unpolluted to moderately polluted (I_geo_ = 0–1) status for V, Mn, Co, Ni, Zn, Sr, Cd, Ba, and Tl. Moderately polluted (Igeo = 1–2) and moderately to strongly polluted (I_geo_ = 2–3) levels were observed for Cr, Fe, Cu, As, Sb, Sn, and Pb, as shown in Table S3. For most metals, except Cr, Fe, Cu, As, Sb, Sn, and Pb, the I_geo_ classes across all sampling stations ranged between 0 and 1, suggesting that these areas are largely uncontaminated by these metals. However, I_geo_ values reached moderately to strongly polluted levels for some heavy metals at sampling stations PG1, PG3, PG4, and PG10, with the highest I_geo_ value of 2.560 for Pb, indicating moderate to strong pollution. Overall, the I_geo_ levels for the studied metals follow this order: Pb > Cr > As > Cu > Fe > Sn > Sb.

### Enrichment factor (EF)

The enrichment factor (EF) index revealed that Sb had the highest contamination level (616) among the metals studied in surface dust samples at the PG1 site, classifying it as extreme contamination. Similarly, As, Cd, Sb, and Tl showed extreme EF values (EF > 40). According to the EF index, V, Cr, Mn, Fe, Co, Ni, Zn, Sr, and Ba fall into the minimal contamination category (EF < 2); Cu and Sn are in the moderate contamination category (EF = 2–5); and Pb is in the significant contamination category (EF = 5–20) (Table S4).

According to some researchers, an EF value between 0.05 and 1.5 suggests that the metals originate entirely from the Earth's crust or natural processes, while an EF > 1.5 indicates a manmade origin (Kamani et al. 2017; Mohseni-Bandpei et al., 2017). Thus, V, Cr, Mn, Fe, Co, Ni, Zn, Sr, and Ba likely have a natural origin, while human activities have increased the concentrations of As, Cd, Cu, Zn, Sb, Tl, and Pb at the sampling sites. The mean EF index for the studied metals follows this trend: Tl < As < Cd < Sb.

### Ecological risk factor (Er_i_) and potential ecological risk (RI)

Using the formulas reported by Hakanson (1980), the ecological risk index (Er_i_) and potential ecological risk index (RI) levels were calculated, with results provided in Table S5. All elements, except As and Pb, showed low ecological risk levels. Moderate ecological risk levels for As and Pb were found in surface dust samples from the PG4 site. Most values indicated low to moderate ecological risk. Low potential ecological risk levels (RI < 150) were found at PG2, PG3, PG5, PG6, PG7, PG8, PG10, and PG11. A moderate potential ecological risk (RI = 150–300) was found only at PG1 and PG4, primarily due to the moderate ecological risk posed by As and Pb in these districts.

An assessment of the strengths and limitations of this study was made, and the following results were obtained: This study involved extensive data collection from multiple children's playgrounds, ensuring a robust dataset for analysis. Utilizing well-recognized geochemical indices like the geoaccumulation index (I_geo_) and enrichment factor (EF) provides reliable and comparable results. The inclusion of various HMs and the use of multiple indicators and statistical analyses offer a comprehensive understanding of the contamination levels and associated risks. Focusing on children’s playgrounds highlights a significant public health concern, addressing a vulnerable population. Incorporating a detailed risk assessment for HM exposure provides actionable insights for public health and environmental policy.

The study is limited to playgrounds within a specific radius of a cement factory in Bursa, Türkiye, which may not be representative of other areas. The absence of pre-industrial values necessitated the use of continental crust values for I_geo_ calculations, which might not perfectly reflect historical contamination levels. The number of playgrounds sampled may limit the generalizability of the findings to other urban areas. Differences in children's play habits and time spent in playgrounds were not accounted for, which could affect individual exposure levels. The study provides a snapshot in time and does not account for seasonal or long-term variations in HM concentrations.

HMs enter the body through inhaling contaminated air, skin absorption, consuming contaminated food, and drinking contaminated water. Children's exposure to HMs adversely affects their health through various mechanisms, depending on the specific metal. Mercury, lead, chromium, cadmium, and barium pose particularly urgent health concerns due to their widespread use, high toxicity, and prevalence. The adverse effects of HM toxicity vary, but many HMs share common harmful health impacts. For instance, many of these metals cause mental retardation and neurocognitive disorders in children, such as impaired memory and lower IQ, which hinder academic performance and cause behavioral disorders. Additionally, mercury, lead, and cadmium impair children's growth. Chromium and cadmium are known to cause cancers, including sinus, lung, and nasal cancers, while mercury and chromium are linked to diabetes. HM poisoning also leads to respiratory issues, such as airway irritation, obstruction, and diseases like rhinitis, bronchitis, and asthma. Furthermore, they contribute to cardiovascular diseases, including heart failure, stroke, coronary heart disease, and abnormal blood pressure. Given the detrimental health effects of heavy metal poisoning, efforts should be made to reduce their pollution and environmental release, achievable through stringent laws and regulations (Zaynab et al., 2022).

## Conclusions

In this study, HM concentration levels in surface dust in Bursa and the health risks that children may be exposed to playing in playgrounds were investigated. Based on the findings, the concentrations of Cr, Pb, Cu and Ni were in the range of 2.07 to 232.46, 0.07 to 19.07, 0.74 to 51.71, and 7.97 to 81.64 mg/kg, respectively. Fe and Zn emerged as the predominant heavy metal species in the samples. The metal concentrations followed a descending order at the PG4 > PG3 > PG1 > PG2 > PG5 > PG6 > PG8 > PG11 > PG10 > PG9 > PG7 sites, indicating an increase in concentration levels nearer to the factory due to heavy metal-containing dust deposition. Conversely, concentrations decreased with distance from the source. Multivariate statistical analysis suggested that Mn, Fe, Co, Cu, Zn, Cd, As, and Ni primarily originate from anthropogenic sources, while Cr and Pb levels may be influenced by vehicular emissions. Cement production processes and vehicular emissions were identified as the principal sources of these metals in the studied areas.

HQ and HI calculations were conducted for children across three exposure routes, revealing that HQ values for all analyzed HMs remained below the safe threshold (HQ < 1), and non-carcinogenic health risks from individual heavy metals in surface dust samples were within acceptable safety limits. Cancer risk levels associated with carcinogenic HMs fell within the safety range provided by USEPA, indicating a negligible likelihood of carcinogenesis due to metals in the surface dust samples studied. The exposure routes for children generally showed an order of ingestion > skin contact > inhalation in most samples, emphasizing the importance of hand and face washing to mitigate exposure. Given children's increased vulnerability to surface dust pollutants due to heightened hand-to-mouth activities and weaker immunity compared to adults, these findings underscore the necessity of protective measures.

The acquired data were used to generate the Geoaccumulation Index (I_geo_), Enrichment Factor (EF), Ecological Risk Factor (Er_i_), and Potential Ecological Risk (RI). Most metals, with the exception of Cr, Fe, Cu, As, Sb, Sn, and Pb, were found to have I_geo_ classifications ranging from 0 to 1, suggesting that these areas are mainly uncontaminated. The mean EF index for the examined metals was as follows: Tl < As < Cd < Sb. Except for As and Pb, all elements had minor ecological risks. Surface dust tests from the PG4 site contained moderate amounts of As and Pb, both of which pose ecological risks. Overall, most results suggested a low to moderate environmental risk.

Children are more sensitive to chemical exposure as their organs are still developing and not yet mature. With the globalization of environmental pollution and the rapid economic growth, the exposure of low concentrations of heavy metals in children is increasingly a global health concern. Understanding the concentrations and sources of HMs in children's playgrounds is vital for developing effective strategies to mitigate exposure and protect public health. The health risks associated with HM contamination are substantial, particularly for children, necessitating comprehensive and proactive measures to ensure safe and healthy play environments.

Future research should prioritize the following areas to better understand and mitigate the risks associated with heavy metal contamination:

- Continuous monitoring of HM concentrations in various urban environments, especially in children's playgrounds, to detect trends and changes over time.
- Evaluation of the effectiveness of different remediation techniques for reducing HM levels in contaminated soils and dust. This includes the use of plants (phytoremediation), soil amendments, and technological interventions.
- In-depth studies on the health impacts of chronic low-level exposure to HMs, particularly in children, This involves both epidemiological studies and clinical research to understand the full extent of health effects.
- Research into the specific pathways through which children are exposed to HMs, including ingestion, inhalation, and dermal absorption, is needed to develop targeted interventions.
- Analysis of the effectiveness of current policies and regulations related to HM emissions and contamination. Recommendations for new policies or revisions to existing ones to better protect public health.
- Studies on the best methods for educating communities about the risks of HM exposure and ways to minimize them. This includes public health campaigns and community-led monitoring programs.
- Development and implementation of advanced technologies for detecting and quantifying HMs in various environmental matrices (soil, water, and air).
- Comparative studies between different geographic regions are needed to understand how local industrial activities, urbanization patterns, and environmental regulations impact HM contamination levels.
- Investigation into how genetic and epigenetic factors influence susceptibility to HM toxicity could lead to personalized approaches to prevention and treatment.
- Development of integrated approaches that combine environmental science, public health, and urban planning to create healthier urban environments.

By addressing these areas, future research can contribute significantly to reducing HM exposure and its associated health risks, particularly in vulnerable populations such as children.

## Supplementary Information

Below is the link to the electronic supplementary material.

Supplementary file1 (PDF 1112 kb)

## Data Availability

No datasets were generated or analysed during the current study.
