# Supplementary material for: Assessing heavy metal contamination and health risks in playground dust near cement factory: exposure levels in children
Source: Environ Geochem Health. 2024 Aug 21;46(10):368. doi: 10.1007/s10653-024-02144-7 (PMC11339112; doi:10.1007/s10653-024-02144-7)
Supplement: Supplementary file 1 — Supplementary file1 (PDF 1112 kb) [file 10653_2024_2144_MOESM1_ESM.pdf]

Assessing Heavy Metal Contamination and Health Risks in Playground Dust Near Cement Factory: Exposure Levels in Children

Aşkın BİRGÜL[1*]

[1]Bursa Technical University, Faculty of Engineering and Natural Sciences, Department of Environmental Engineering, Mimar Sinan Mahallesi Mimar Sinan Bulvarı Eflak Caddesi No:177 16310 Yıldırım/Bursa-TÜRKİYE

*Corresponding author, e-mail: askin.birgul@btu.edu.tr Tel: (+90) 2243003484; Fax: (+90) 2243003419

Aşkın BİRGÜL: https://orcid.org/0000-0002-7718-0340

Supplementary Material

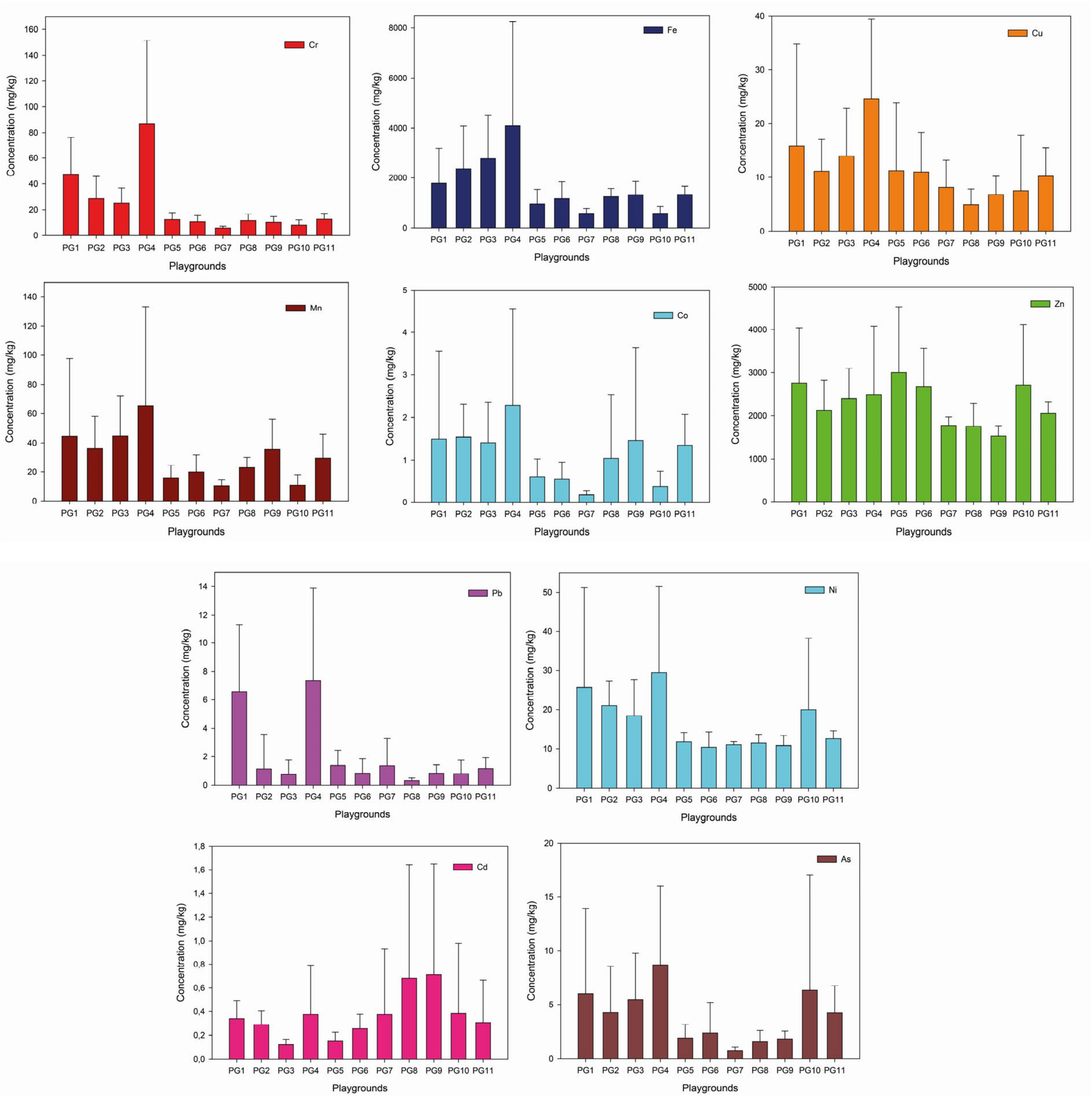

Fig. S1 Concentration levels of individual HMs

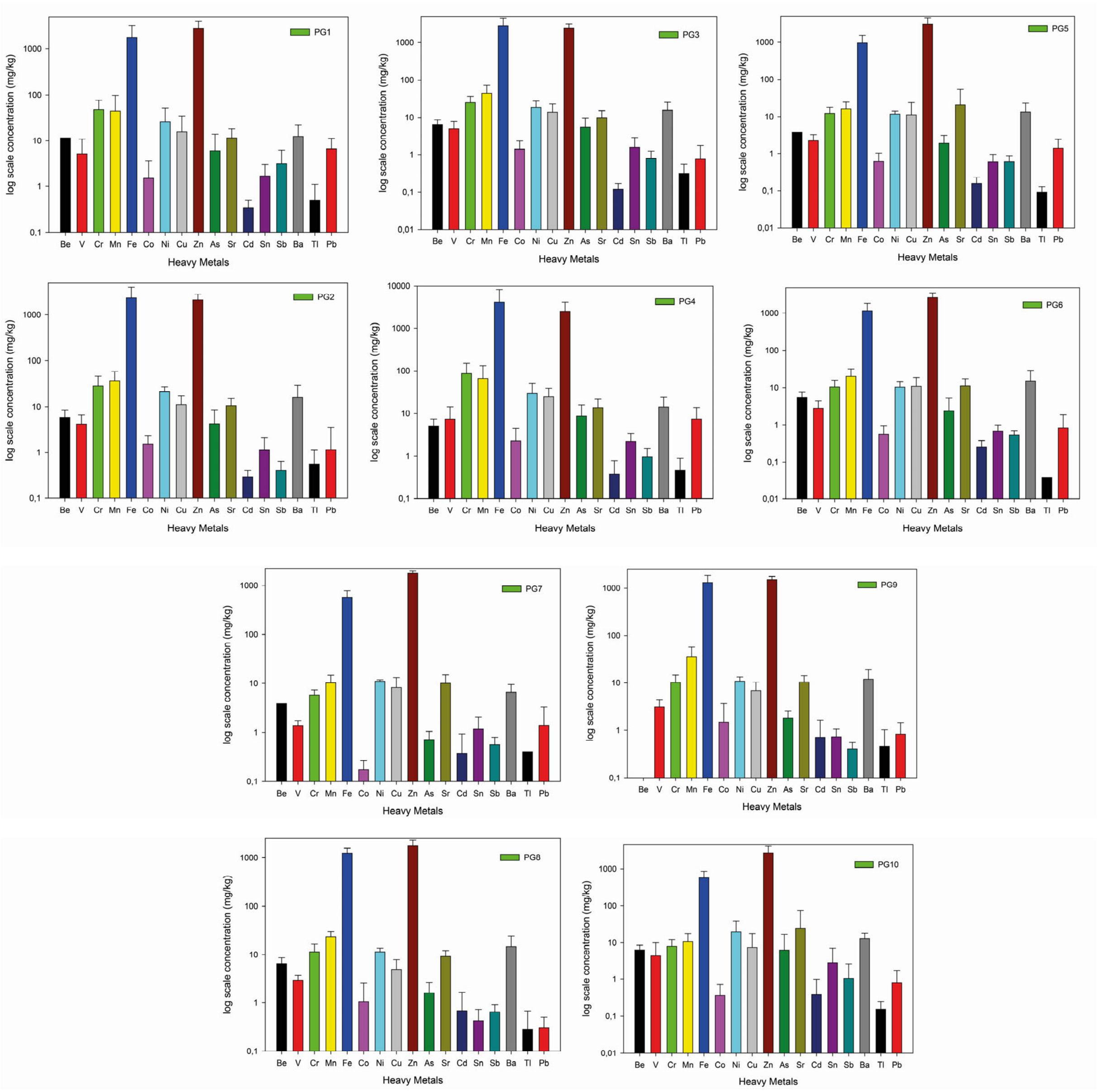

Fig. S2 HM concentration levels at the sampling sites

Table S1 Descriptive statistics of heavy metal concentrations (mg/kg) in surface dust samples around cement factory.

PG1	V	Cr	Mn	Fe	Co	Ni	Cu	Zn	As	Sr	Cd	Sn	Sb	Ba	Tl	Pb	Total
PG01-01	1.32	51.14	5.07	285.89	0.11	<MDL	1.97	1661.96	nd	3.99	nd	1.04	3.58	7.50	nd	12.36	2035.94
PG01-02	4.33	50.84	36.17	2643.85	1.12	12.40	9.88	1393.62	2.66	7.03	0.34	0.91	1.37	6.94	0.24	6.02	4177.71
PG01-03	1.73	8.84	12.86	744.77	0.36	<MDL	2.59	1575.04	0.48	3.04	0.09	0.39	0.39	4.50	0.05	nd	2355.13
PG01-04	3.86	27.32	29.20	1838.62	1.02	14.67	7.73	1605.64	5.08	7.08	nd	0.60	<MDL	3.56	0.19	nd	3544.56
PG01-05	20.28	113.91	183.49	1337.86	7.03	81.64	49.33	3148.52	26.37	22.52	0.51	4.84	1.37	15.37	1.82	11.61	5026.47
PG01-06	2.10	22.65	24.67	1081.33	0.63	10.24	6.25	5228.37	2.66	9.99	nd	0.91	1.37	5.34	0.06	1.52	6398.11
PG01-07	2.89	53.17	19.08	1199.45	0.54	10.63	6.60	4030.46	1.21	10.29	0.43	2.12	5.72	16.03	0.15	9.53	5368.29
PG01-08	0.94	61.00	6.66	439.12	0.19	<MDL	2.71	3751.26	2.42	15.27	nd	1.77	10.27	8.44	nd	9.00	4309.05
PG01-09	7.19	53.74	62.99	4227.92	1.92	27.85	19.17	2551.61	5.56	17.20	0.43	2.12	1.53	25.13	0.80	1.55	7006.70
PG01-10	6.50	31.22	64.61	4004.11	2.05	22.04	51.71	2669.32	7.50	18.74	0.26	2.03	2.15	31.50	0.63	1.01	6915.37
**Mean**	5.11	47.38	44.48	1780.29	1.50	25.64	15.79	2761.58	5.99	11.52	0.34	1.67	3.08	12.43	0.49	6.58	**Mean±sd**
**sd**	5.73	28.81	53.12	1406.78	2.06	25.53	18.97	1278.08	7.96	6.60	0.15	1.29	3.13	9.48	0.60	4.71	4713.73±1764.46
**Max**	20.28	113.91	183.49	4227.92	7.03	81.64	51.71	5228.37	26.37	22.52	0.51	4.84	10.27	31.50	1.82	12.36	
**Min**	0.94	8.84	5.07	285.89	0.11	10.24	1.97	1393.62	0.48	3.04	0.09	0.39	0.39	3.56	0.05	1.01	
**n**	10.00	10.00	10.00	10.00	10.00	7.00	10.00	10.00	9.00	10.00	6.00	10.00	9.00	10.00	8.00	8.00	
**PG2**	**V**	**Cr**	**Mn**	**Fe**	**Co**	**Ni**	**Cu**	**Zn**	**As**	**Sr**	**Cd**	**Sn**	**Sb**	**Ba**	**Tl**	**Pb**	**Total**
PG02-01	0.85	3.62	2.67	144.47	nd	<MDL	2.90	1551.54	nd	4.25	nd	0.35	nd	7.22	nd	0.11	1717.98
PG02-02	6.37	51.01	62.95	5693.69	1.56	20.26	19.06	1425.25	6.53	9.44	0.43	0.86	<MDL	6.66	0.47	0.25	7304.79
PG02-03	1.91	13.79	18.98	948.66	0.34	<MDL	3.54	1333.45	0.73	5.10	nd	0.22	0.20	4.59	nd	<MDL	2331.52
PG02-04	4.77	39.30	41.22	2507.91	1.56	18.93	10.90	1651.96	2.90	12.31	0.34	0.56	<MDL	5.81	0.24	0.07	4298.80
PG02-05	8.54	55.44	75.25	4750.63	3.03	34.61	20.78	3055.32	14.51	19.17	0.34	2.07	0.81	21.75	1.73	7.56	8071.55
PG02-06	3.20	21.39	30.79	1761.03	2.19	16.66	11.08	3169.98	2.42	12.78	0.17	2.12	0.16	11.16	0.09	0.44	5045.66
PG02-07	4.17	31.13	43.31	2763.20	1.85	20.37	15.71	2734.93	5.08	9.05	0.17	2.89	0.62	15.84	0.33	1.00	5649.65
PG02-08	3.96	23.68	29.71	1829.39	1.00	16.28	7.28	2522.99	3.14	12.52	nd	0.47	0.33	15.09	0.30	0.16	4466.30
PG02-09	6.12	36.36	42.54	2279.60	1.33	19.65	9.45	2224.47	2.42	13.34	nd	1.60	0.23	50.73	0.79	0.56	4689.19
PG02-10	1.79	8.95	13.87	910.61	0.99	<MDL	10.42	1538.61	0.48	7.38	nd	0.17	0.49	19.03	<MDL	0.18	2512.96

**Mean**	4.17	28.47	36.13	2358.92	1.54	20.97	11.11	2120.85	4.25	10.53	0.29	1.13	0.40	15.79	0.57	1.15	**Mean± sd**
**sd**	2.38	17.35	21.98	1721.14	0.78	6.23	5.97	707.91	4.30	4.44	0.11	0.96	0.24	13.62	0.56	2.42	4608.84±2070.74
**Max**	8.54	55.44	75.25	5693.69	3.03	34.61	20.78	3169.98	14.51	19.17	0.43	2.89	0.81	50.73	1.73	7.56	
**Min**	0.85	3.62	2.67	144.47	0.34	16.28	2.90	1333.45	0.48	4.25	0.17	0.17	0.16	4.59	0.09	0.07	
**n**	10.00	10.00	10.00	10.00	9.00	7.00	10.00	10.00	9.00	10.00	5.00	10.00	7.00	10.00	7.00	9.00	
**PG3**	**V**	**Cr**	**Mn**	**Fe**	**Co**	**Ni**	**Cu**	**Zn**	**As**	**Sr**	**Cd**	**Sn**	**Sb**	**Ba**	**Tl**	**Pb**	**Total**
PG03-01	2.04	9.44	11.67	703.53	0.34	<MDL	2.85	1610.27	nd	4.85	nd	<MDL	nd	7.12	nd	0.10	2352.22
PG03-02	7.41	35.93	57.53	4333.20	1.93	20.04	15.26	1589.42	5.32	5.40	nd	0.39	0.68	6.94	0.11	0.21	6079.78
PG03-03	2.45	12.63	22.70	1214.30	0.40	8.36	5.69	1790.39	nd	4.50	0.17	1.12	nd	6.56	<MDL	0.21	3069.49
PG03-04	1.88	16.71	23.34	1494.05	0.92	11.46	5.27	1683.37	0.73	5.79	nd	0.30	0.39	5.25	nd	<MDL	3249.45
PG03-05	9.45	37.76	86.22	5055.00	3.03	33.22	22.91	3121.55	12.82	17.16	0.09	2.63	1.14	24.94	0.41	3.08	8431.40
PG03-06	8.48	38.63	89.61	5405.09	2.75	31.45	24.75	2347.09	7.74	9.78	0.09	3.97	1.59	14.34	0.65	0.79	7986.80
PG03-07	5.40	33.92	55.64	3929.39	1.80	21.76	18.77	2646.25	9.92	8.28	nd	3.11	0.68	27.09	0.31	1.12	6763.43
PG03-08	2.35	15.90	21.21	1183.99	0.74	10.69	6.14	2955.56	2.18	10.51	0.17	0.39	<MDL	9.84	0.05	nd	4219.72
PG03-09	6.06	33.86	45.85	2538.73	1.46	17.83	10.92	3505.67	2.66	18.06	nd	1.12	0.16	30.10	0.55	0.23	6213.25
PG03-10	4.11	14.66	32.65	1796.05	0.69	10.85	26.57	2747.25	2.42	15.27	0.09	1.08	0.78	23.91	0.11	0.27	4676.75
**Mean**	4.96	24.94	44.64	2765.33	1.41	18.41	13.91	2399.68	5.47	9.96	0.12	1.57	0.78	15.61	0.31	0.75	**Mean± sd**
**sd**	2.82	11.92	27.39	1757.16	0.96	9.15	8.93	699.34	4.30	5.19	0.05	1.34	0.47	9.83	0.23	1.01	5304.23±2109.05
**Max**	9.45	38.63	89.61	5405.09	3.03	33.22	26.57	3505.67	12.82	18.06	0.17	3.97	1.59	30.10	0.65	3.08	
**Min**	1.88	9.44	11.67	703.53	0.34	8.36	2.85	1589.42	0.73	4.50	0.09	0.30	0.16	5.25	0.05	0.10	
**n**	10.00	10.00	10.00	10.00	10.00	9.00	10.00	10.00	8.00	10.00	5.00	9.00	7.00	10.00	7.00	8.00	
**PG4**	**V**	**Cr**	**Mn**	**Fe**	**Co**	**Ni**	**Cu**	**Zn**	**As**	**Sr**	**Cd**	**Sn**	**Sb**	**Ba**	**Tl**	**Pb**	**Total**
PG04-01	1.07	79.79	3.04	208.10	nd	<MDL	<MDL	1482.29	nd	4.33	nd	1.47	nd	5.16	nd	10.29	1795.53
PG04-02	8.38	150.29	62.07	5380.44	1.95	21.92	19.08	1518.01	4.84	7.42	0.26	1.81	<MDL	5.16	0.49	19.70	7201.82
PG04-03	1.10	5.71	11.06	589.88	0.29	<MDL	37.99	792.88	nd	5.45	nd	1.04	0.16	3.94	nd	2.52	1452.00
PG04-04	3.83	95.61	33.84	1976.12	0.92	15.06	7.69	1530.80	3.87	6.69	nd	1.34	0.46	4.69	0.07	4.26	3685.22
PG04-05	14.91	70.56	155.37	9456.72	5.40	57.54	39.79	3048.81	21.29	24.32	0.17	3.80	1.63	17.34	0.77	5.36	12923.76

PG04-06	23.52	81.14	212.04	12787.72	6.50	69.06	49.33	5340.44	19.11	23.12	1.11	3.50	1.07	19.22	1.39	1.72	18639.97
PG04-07	2.76	22.78	30.79	1699.80	0.97	13.79	9.97	4335.72	3.87	12.44	nd	2.12	0.94	17.91	0.16	1.38	6155.39
PG04-08	9.48	53.24	82.87	5176.09	3.21	31.45	17.87	4063.00	7.98	19.69	0.26	0.91	1.20	12.56	0.35	1.01	9481.16
PG04-09	3.99	75.09	29.88	1647.27	0.56	12.95	11.95	1745.00	1.94	11.84	nd	2.46	0.46	25.78	0.22	12.75	3582.12
PG04-10	4.30	232.46	34.01	1993.68	0.82	13.84	27.64	1000.69	6.53	22.95	0.09	3.93	1.79	31.69	0.27	14.42	3389.09
**Mean**	7.33	86.67	65.50	4091.58	2.29	29.45	24.59	2485.76	8.68	13.82	0.38	2.24	0.96	14.34	0.46	7.34	**Mean± sd**
**sd**	7.12	64.64	67.66	4152.11	2.27	21.99	14.86	1593.45	7.36	7.97	0.42	1.14	0.58	9.72	0.43	6.54	6830.61±5485.11
**Max**	23.52	232.46	212.04	12787.72	6.50	69.06	49.33	5340.44	21.29	24.32	1.11	3.93	1.79	31.69	1.39	19.70	
**Min**	1.07	5.71	3.04	208.10	0.29	12.95	7.69	792.88	1.94	4.33	0.09	0.91	0.16	3.94	0.07	1.01	
**n**	10.00	10.00	10.00	10.00	9.00	8.00	9.00	10.00	8.00	10.00	5.00	10.00	8.00	10.00	8.00	10.00	
**PG5**	**V**	**Cr**	**Mn**	**Fe**	**Co**	**Ni**	**Cu**	**Zn**	**As**	**Sr**	**Cd**	**Sn**	**Sb**	**Ba**	**Tl**	**Pb**	**Total**
PG05-01	1.38	5.47	4.26	223.75	0.11	<MDL	0.74	1609.39	nd	4.25	nd	0.13	nd	5.91	nd	<MDL	1855.38
PG05-02	3.14	18.80	27.07	2114.69	0.69	11.57	9.97	1455.09	1.69	5.15	nd	0.30	nd	3.09	0.09	nd	3651.34
PG05-03	0.88	5.20	7.00	339.16	0.20	<MDL	1.45	2306.59	nd	6.00	0.09	0.43	<MDL	6.37	nd	nd	2673.39
PG05-04	2.07	10.93	10.89	743.95	0.83	<MDL	7.53	2473.26	0.48	6.43	0.17	0.69	0.49	6.19	nd	0.46	3264.39
PG05-05	3.99	18.19	28.49	1578.03	1.02	14.73	23.12	4318.05	4.11	16.86	0.17	1.30	0.98	9.75	0.11	2.24	6021.12
PG05-06	2.32	16.58	22.53	1141.31	1.35	11.85	10.22	6381.24	1.21	10.25	nd	0.78	0.55	18.38	0.09	0.65	7619.30
PG05-07	2.42	14.72	14.65	944.52	0.86	<MDL	8.31	4142.30	0.97	10.46	nd	0.73	0.52	13.12	0.04	0.57	5154.19
PG05-08	1.63	13.02	16.58	718.72	0.13	<MDL	4.82	2138.88	1.94	114.68	0.26	0.17	0.36	12.61	nd	2.96	3026.77
PG05-09	1.13	5.29	6.77	442.15	0.21	<MDL	3.64	2167.78	nd	11.11	nd	0.47	0.29	26.16	nd	0.50	2665.51
PG05-10	3.55	14.40	21.31	1247.99	0.63	8.80	42.33	3016.85	2.90	19.26	0.09	0.86	0.98	32.44	0.13	2.30	4414.81
**Mean**	2.25	12.26	15.96	949.43	0.60	11.74	11.21	3000.95	1.90	20.44	0.15	0.59	0.59	13.40	0.09	1.38	**Mean± sd**
**sd**	1.04	5.31	8.70	589.80	0.42	2.42	12.64	1527.09	1.24	33.48	0.07	0.36	0.28	9.59	0.03	1.07	4034.62±1777.22
**Max**	3.99	18.80	28.49	2114.69	1.35	14.73	42.33	6381.24	4.11	114.68	0.26	1.30	0.98	32.44	0.13	2.96	
**Min**	0.88	5.20	4.26	223.75	0.11	8.80	0.74	1455.09	0.48	4.25	0.09	0.13	0.29	3.09	0.04	0.46	
**n**	10.00	10.00	10.00	10.00	10.00	4.00	10.00	10.00	7.00	10.00	5.00	10.00	7.00	10.00	5.00	7.00	
**PG6**	**V**	**Cr**	**Mn**	**Fe**	**Co**	**Ni**	**Cu**	**Zn**	**As**	**Sr**	**Cd**	**Sn**	**Sb**	**Ba**	**Tl**	**Pb**	**Total**

PG06-02	3.30	16.95	24.94	1596.39	1.13	9.52	8.73	3254.83	1.94	3.95	nd	0.52	nd	33.85	nd	0.21	4956.24
PG06-03	0.91	3.83	8.80	482.13	0.09	<MDL	1.96	1473.51	nd	3.56	nd	0.52	<MDL	3.47	nd	nd	1978.76
PG06-04	2.07	6.08	11.71	637.89	0.14	<MDL	5.27	1953.38	nd	7.68	nd	0.30	0.46	2.53	nd	nd	2627.49
PG06-05	5.84	18.69	45.04	2595.90	1.06	17.33	17.95	3069.20	8.71	20.46	0.34	1.38	0.78	8.16	0.04	2.87	5813.74
PG06-06	1.79	8.69	13.97	863.02	0.37	<MDL	10.61	2826.61	0.73	16.13	nd	0.43	0.46	2.16	nd	0.29	3745.24
PG06-07	2.95	13.39	15.73	1218.32	0.41	9.02	11.42	2530.32	1.45	10.81	0.17	0.95	0.46	11.53	nd	0.61	3827.55
PG06-08	1.41	6.84	12.04	591.79	0.23	<MDL	2.57	4527.73	0.97	11.41	nd	0.47	0.39	7.69	nd	nd	5163.55
PG06-09	4.46	10.32	31.57	1587.21	0.94	8.08	15.69	2142.20	1.94	10.68	nd	0.65	0.72	24.28	nd	0.31	3839.04
PG06-10	2.39	9.95	15.60	908.40	0.56	8.08	24.48	2352.54	0.73	16.43	nd	0.73	0.42	39.19	nd	0.70	3380.19
**Mean**	2.79	10.53	19.93	1164.56	0.55	10.41	10.96	2681.15	2.35	11.23	0.26	0.66	0.52	14.76	0.04	0.83	**Mean± sd**
**sd**	1.56	4.98	11.82	674.95	0.40	3.92	7.44	888.82	2.85	5.71	0.12	0.33	0.16	14.11		1.02	3925.76±1223.83
**Max**	5.84	18.69	45.04	2595.90	1.13	17.33	24.48	4527.73	8.71	20.46	0.34	1.38	0.78	39.19	0.04	2.87	
**Min**	0.91	3.83	8.80	482.13	0.09	8.08	1.96	1473.51	0.73	3.56	0.17	0.30	0.39	2.16	0.04	0.21	
**n**	9.00	9.00	9.00	9.00	9.00	5.00	9.00	9.00	7.00	9.00	2.00	9.00	7.00	9.00	1.00	6.00	
**PG7**	**V**	**Cr**	**Mn**	**Fe**	**Co**	**Ni**	**Cu**	**Zn**	**As**	**Sr**	**Cd**	**Sn**	**Sb**	**Ba**	**Tl**	**Pb**	**Total**
PG07-01	1.73	8.15	12.08	619.78	0.20	11.18	10.22	1777.66	1.21	8.62	nd	3.32	0.42	3.00	nd	nd	2457.58
PG07-02	1.19	4.40	9.64	448.59	0.04	<MDL	2.96	1582.99	nd	3.60	nd	0.60	0.62	3.56	nd	nd	2058.20
PG07-04	1.22	4.33	5.78	277.60	nd	<MDL	3.19	1668.98	0.73	6.78	nd	0.30	0.36	8.34	nd	nd	1977.62
PG07-05	1.76	7.27	18.40	880.19	0.26	11.68	11.97	2012.86	0.24	20.76	0.17	1.08	0.88	7.22	nd	0.06	2974.79
PG07-06	0.66	4.86	7.78	368.80	0.07	<MDL	5.69	1496.46	0.73	10.98	0.17	1.47	0.19	7.12	nd	1.85	1906.83
PG07-07	1.76	6.97	16.17	814.51	0.21	<MDL	14.39	2018.71	0.48	10.89	nd	0.86	0.85	7.87	nd	0.13	2893.80
PG07-08	1.16	4.20	5.89	450.70	<MDL	<MDL	4.72	1839.68	nd	9.39	0.09	0.82	0.65	12.00	nd	nd	2329.30
PG07-09	1.63	5.14	9.57	669.84	0.29	<MDL	4.38	1962.71	nd	13.12	0.09	0.52	nd	3.03	nd	0.32	2670.63
PG07-10	1.11	6.66	6.89	654.48	0.13	10.11	15.81	1624.20	0.89	6.40	1.36	1.49	0.51	6.68	0.40	4.51	2341.65
**Mean**	1.36	5.78	10.25	576.05	0.17	10.99	8.15	1776.03	0.71	10.06	0.38	1.16	0.56	6.54	0.40	1.37	**Mean± sd**
**sd**	0.38	1.49	4.50	203.17	0.09	0.80	5.00	194.88	0.33	4.92	0.55	0.91	0.24	2.94		1.90	2401.16±386.91
**Max**	1.76	8.15	18.40	880.19	0.29	11.68	15.81	2018.71	1.21	20.76	1.36	3.32	0.88	12.00	0.40	4.51	
**Min**	0.66	4.20	5.78	277.60	0.04	10.11	2.96	1496.46	0.24	3.60	0.09	0.30	0.19	3.00	0.40	0.06	

n	9.00	9.00	9.00	9.00	7.00	3.00	9.00	9.00	6.00	9.00	5.00	9.00	8.00	9.00	1.00	5.00	
**PG8**	**V**	**Cr**	**Mn**	**Fe**	**Co**	**Ni**	**Cu**	**Zn**	**As**	**Sr**	**Cd**	**Sn**	**Sb**	**Ba**	**Tl**	**Pb**	**Total**
PG08-01	2.13	6.53	21.25	1146.55	0.66	<MDL	1.14	1595.98	nd	5.75	nd	0.17	nd	7.12	nd	nd	2787.29
PG08-02	3.58	8.19	29.37	1598.35	0.96	9.25	2.87	1627.92	nd	4.67	nd	0.22	nd	6.19	nd	nd	3291.56
PG08-03	2.26	6.42	14.55	804.39	0.43	<MDL	1.88	2085.47	nd	6.39	nd	0.13	<MDL	6.56	nd	nd	2928.48
PG08-04	1.54	6.25	14.58	787.55	0.19	<MDL	1.18	1965.05	nd	8.19	nd	0.13	<MDL	7.41	nd	<MDL	2792.06
PG08-05	3.36	10.15	23.07	1298.11	0.39	14.06	9.14	1181.07	1.69	12.35	nd	0.82	0.39	19.50	0.05	0.13	2574.29
PG08-06	2.32	8.82	21.99	1260.86	0.52	9.69	5.73	2841.73	0.48	11.88	0.17	0.52	0.42	8.91	<MDL	0.45	4174.49
PG08-07	2.83	19.49	19.15	1215.55	0.62	10.19	8.60	1102.77	1.94	11.15	nd	0.99	0.46	19.31	0.05	0.17	2413.25
PG08-08	3.61	15.40	35.22	1704.39	0.73	10.52	5.60	1982.56	0.48	8.58	nd	0.26	0.65	34.78	nd	0.21	3802.99
PG08-09	3.89	15.97	32.52	1645.76	0.70	11.29	6.22	1180.64	3.14	12.78	0.09	0.65	0.98	20.06	0.17	<MDL	2934.86
PG08-10	3.16	17.04	19.60	1007.35	5.24	15.00	6.46	2010.11	1.79	10.39	1.79	0.30	0.98	17.35	0.87	0.57	3117.97
**Mean**	2.87	11.43	23.13	1246.89	1.04	11.43	4.88	1757.33	1.59	9.21	0.68	0.42	0.65	14.72	0.28	0.31	**Mean± sd**
**sd**	0.77	5.03	7.10	328.29	1.49	2.23	2.95	535.13	1.00	2.92	0.96	0.31	0.27	9.23	0.39	0.19	3081.72±545.66
**Max**	3.89	19.49	35.22	1704.39	5.24	15.00	9.14	2841.73	3.14	12.78	1.79	0.99	0.98	34.78	0.87	0.57	
**Min**	1.54	6.25	14.55	787.55	0.19	9.25	1.14	1102.77	0.48	4.67	0.09	0.13	0.39	6.19	0.05	0.13	
**n**	10.00	10.00	10.00	10.00	10.00	7.00	10.00	10.00	6.00	10.00	3.00	10.00	6.00	10.00	4.00	5.00	
**PG9**	**V**	**Cr**	**Mn**	**Fe**	**Co**	**Ni**	**Cu**	**Zn**	**As**	**Sr**	**Cd**	**Sn**	**Sb**	**Ba**	**Tl**	**Pb**	**Total**
PG09-01	3.23	7.99	36.58	1520.76	0.69	9.08	3.37	1389.53	0.48	4.12	nd	0.13	<MDL	4.97	nd	<MDL	2980.93
PG09-02	4.11	11.28	59.60	2277.09	0.94	11.96	5.92	1669.94	nd	6.78	0.09	nd	0.29	7.87	nd	nd	4055.87
PG09-04	2.67	11.04	21.45	1011.22	0.49	12.24	5.85	1382.28	1.21	15.05	0.26	0.52	0.39	19.88	<MDL	0.34	2484.87
PG09-05	5.24	20.34	54.76	1589.77	1.30	15.27	14.37	1155.54	1.94	14.93	nd	0.86	0.39	20.25	0.05	1.68	2896.69
PG09-06	3.67	9.63	39.83	1643.92	0.94	13.51	5.38	1606.20	2.42	12.40	nd	0.86	0.20	3.84	nd	nd	3342.80
PG09-07	1.32	7.21	6.73	519.29	0.13	8.08	4.12	1963.82	nd	6.09	nd	0.82	0.55	10.41	nd	nd	2528.57
PG09-08	4.11	12.37	64.03	1240.45	0.94	9.19	8.50	1583.88	2.18	12.70	nd	1.25	0.68	15.00	<MDL	0.52	2955.82

PG10-09	2.01	5.42	12.79	700.36	0.50	7.97	4.40	1311.82	2.18	11.02	nd	0.56	0.36	19.88	nd	<MDL	2079.26
PG09-10	1.41	5.70	24.06	1380.45	7.21	10.02	9.08	1683.59	2.34	9.96	1.79	0.83	0.35	5.56	0.86	0.79	3143.99
**Mean**	3.09	10.11	35.54	1320.37	1.46	10.81	6.78	1527.40	1.82	10.34	0.71	0.73	0.40	11.96	0.46	0.83	**Mean± sd**
**sd**	1.34	4.56	20.79	532.03	2.18	2.56	3.42	242.23	0.71	3.92	0.94	0.33	0.15	6.88	0.57	0.59	2940.98±566.80
**Max**	5.24	20.34	64.03	2277.09	7.21	15.27	14.37	1963.82	2.42	15.05	1.79	1.25	0.68	20.25	0.86	1.68	
**Min**	1.32	5.42	6.73	519.29	0.13	7.97	3.37	1155.54	0.48	4.12	0.09	0.13	0.20	3.84	0.05	0.34	
**n**	9.00	9.00	9.00	9.00	9.00	9.00	9.00	9.00	7.00	9.00	3.00	8.00	8.00	9.00	2.00	4.00	
**PG10**	**V**	**Cr**	**Mn**	**Fe**	**Co**	**Ni**	**Cu**	**Zn**	**As**	**Sr**	**Cd**	**Sn**	**Sb**	**Ba**	**Tl**	**Pb**	**Total**
PG10-01	<MDL	2.07	2.33	184.93	nd	<MDL	1.70	1605.66	nd	3.52	nd	<MDL	nd	12.28	nd	nd	1812.49
PG10-02	1.07	6.56	7.14	386.96	0.11	<MDL	2.36	1773.64	nd	5.58	nd	0.60	0.29	7.03	nd	0.11	2191.45
PG10-03	0.94	4.81	6.43	456.09	0.03	<MDL	2.81	1873.02	2.18	3.77	nd	0.30	0.46	6.56	nd	nd	2357.40
PG10-04	1.70	8.35	7.41	522.31	0.21	16.06	4.67	3201.73	0.24	13.08	0.09	5.53	0.20	15.94	nd	0.15	3797.66
PG10-05	8.51	15.83	26.63	876.46	1.17	14.73	9.86	4379.03	30.24	19.30	nd	1.17	1.40	3.09	0.09	0.43	5387.94
PG10-06	1.44	6.49	10.69	564.00	0.14	<MDL	3.08	2272.01	0.24	7.98	nd	0.56	0.39	14.34	nd	nd	2881.37
PG10-07	2.98	12.46	16.41	960.75	0.47	9.25	7.76	5896.05	5.32	8.02	0.09	1.64	0.42	19.03	<MDL	nd	6940.65
PG10-08	<MDL	10.22	2.88	181.01	nd	<MDL	2.19	2206.17	nd	6.13	0.09	1.34	<MDL	15.47	nd	0.89	2426.37
PG10-09	2.01	5.42	12.79	700.36	0.50	7.97	4.40	2311.82	2.18	11.02	nd	0.56	0.36	19.88	nd	<MDL	3079.26
PG10-10	16.83	8.28	13.90	929.26	0.24	51.94	36.15	1639.48	3.87	169.17	1.28	13.60	4.81	14.43	0.22	2.41	2905.88
**Mean**	4.43	8.05	10.66	576.21	0.36	19.99	7.50	2715.86	6.32	24.76	0.38	2.81	1.04	12.81	0.16	0.80	**Mean± sd**
**sd**	5.58	3.98	7.26	286.87	0.37	18.19	10.40	1403.38	10.70	50.97	0.60	4.35	1.57	5.55	0.09	0.95	3378.05±1608.08
**Max**	16.83	15.83	26.63	960.75	1.17	51.94	36.15	5896.05	30.24	169.17	1.28	13.60	4.81	19.88	0.22	2.41	
**Min**	0.94	2.07	2.33	181.01	0.03	7.97	1.70	1605.66	0.24	3.52	0.09	0.30	0.20	3.09	0.09	0.11	
**n**	8.00	10.00	10.00	10.00	8.00	5.00	10.00	10.00	7.00	10.00	4.00	9.00	8.00	10.00	2.00	5.00	
**PG11**	**V**	**Cr**	**Mn**	**Fe**	**Co**	**Ni**	**Cu**	**Zn**	**As**	**Sr**	**Cd**	**Sn**	**Sb**	**Ba**	**Tl**	**Pb**	**Total**
PG11-01	2.07	4.33	11.84	610.00	0.34	<MDL	1.51	1706.65	1.94	5.92	<MDL	0.52	<MDL	1.31	<MDL	<MDL	2346.43

PG11-02	7.13	14.83	24.62	1239.10	1.46	12.62	7.92	1592.24	1.94	5.40	0.26	0.47	0.07	8.53	<MDL	<MDL	2916.60
PG11-05	8.91	16.49	28.02	1173.19	2.30	13.48	14.34	2240.68	7.66	9.17	0.17	1.49	1.33	7.31	0.10	1.87	3526.51
PG11-06	9.07	16.45	30.11	1531.34	1.56	14.67	19.66	2142.08	6.69	8.28	0.09	2.29	1.01	11.53	0.03	0.46	3795.31
PG11-07	4.43	10.54	23.21	1636.42	0.60	10.91	11.41	2329.44	7.26	9.60	<MDL	3.24	0.98	9.00	<MDL	1.10	4058.11
PG11-08	3.26	9.15	28.25	1392.60	0.67	10.80	8.77	2305.43	2.42	9.09	<MDL	0.56	0.46	8.50	<MDL	0.10	3780.06
PG11-09	9.54	16.99	67.29	1454.77	1.90	15.39	8.43	2098.71	3.14	19.73	0.09	1.25	1.24	7.38	0.03	1.63	3707.51
PG11-10	4.60	11.56	24.17	1597.73	1.93	10.01	9.77	2020.71	2.74	12.98	0.94	1.62	0.85	8.88	0.02	1.86	3710.39
**Mean**	6.13	12.54	29.69	1329.39	1.35	12.55	10.23	2054.49	4.22	10.02	0.31	1.43	0.85	7.81	0.04	1.17	**Mean± sd**
**sd**	2.90	4.47	16.19	333.14	0.72	2.07	5.27	272.20	2.51	4.57	0.36	0.97	0.45	2.93	0.04	0.75	3480.11±564.81
**Max**	9.54	16.99	67.29	1636.42	2.30	15.39	19.66	2329.44	7.66	19.73	0.94	3.24	1.33	11.53	0.10	1.87	
**Min**	2.07	4.33	11.84	610.00	0.34	10.01	1.51	1592.24	1.94	5.40	0.09	0.47	0.07	1.31	0.02	0.10	
**n**	8.00	8.00	8.00	8.00	8.00	7.00	8.00	8.00	8.00	8.00	5.00	8.00	7.00	8.00	4.00	6.00	

Table S2 Comparison of HM levels detected in the current study to results of the studies conducted in other parts of the world (mg/kg)

Country	City	Sample Type	V	Cr	Mn	Fe	Co	Ni	Cu	Zn	As	Sr	Cd	Sn	Sb	Ba	Tl	Pb	References
Türkiye	Kütahya	Soil	na	31.8	na	na	na	57.2	17.9	67.9	18.9	na	0.280	na	na	na	na	27.1	Özkul, 2019
Türkiye	İstanbul	Soil	60.9	61.6	na	na	16.9	31.4	25.7	154	na	na	na	na	na	na	na	33.9	Ak et al. 2012
Poland	Lublin	Soil	na	201	na	na	na	12.0	15.5	78.3	na	na	4.50	na	na	na	na	38.2	Zgłobicki et al. 2021
Saudi Arabia	Riyadh	Soil	na	3.09	na	na	1.87	4.43	4.89	20.9	na	na	0.038	na	na	na	na	1.94	Alotaibi et al. 2006
Spain	Madrid	Soil-2002	12.0	18.0	na	na	3.40	6.50	19.0	74.0	7.10	49.0	0.200	na	0.950	na	0.200	35.0	De Miguel et al. 2007
		Soil-2003	9.50	16.0	na	na	3.20	5.20	12.0	46.0	6.70	43.0	0.100	na	0.600	na	0.100	17.0	
Türkiye	Ordu	Soil	na	21.0	na	na	na	20	25.0	56.0	na	na	na	na	na	na	na	14.0	Parlak et al. 2022
		Dust	na	16.0	na	na	na	13	18.0	26.0	na	na	na	na	na	na	na	8.00	
Türkiye	Ordu	Soil	na	35.9	na	na	na	21.1	43.8	167	na	na	1.69	na	na	na	na	25.4	Yesil and Yesil 2019
		Dust	na	35.3	na	na	na	11.9	na	201	na	na	2.65	na	na	na	na	32.7	
Poland	Rabka Zdrój	Soil	na	na	na	na	10	15.5	7.75	105	0.11	na	2.3	1.5	na	na	na	40	Kicińska 2016
		Dust	na	na	na	na	11	6.8	0.6	11.2	0.24	na	2	2.2	na	na	na	30.8	
China	Hong Kong	Soil	na	na	na	na	na	na	28.4	237	na	na	2.30	na	na	na	na	195	Chen et al. 1997
		Dust	na	na	na	na	na	na	201	1517	na	na	2.63	na	na	na	na	302	
China	Hong Kong	Dust	na	263	na	na	na	na	143	1883	na	na	7.00	na	na	na	na	77.3	Ng et al. 2003
Türkiye	Bursa-PG1	Surface Dust	5.11	47.38	44.48	1780.29	1.50	25.64	15.79	2761.58	5.99	11.52	0.34	1.67	3.08	12.43	0.49	6.58	This study
	Bursa-PG2		4.17	28.47	36.13	2358.92	1.54	20.97	11.11	2120.85	4.25	10.53	0.29	1.13	0.40	15.79	0.57	1.15	
	Bursa-PG3		4.96	24.94	44.64	2765.33	1.41	18.41	13.91	2399.68	5.47	9.96	0.12	1.57	0.78	15.61	0.31	0.75	
	Bursa-PG4		7.33	86.67	65.50	4091.58	2.29	29.45	24.59	2485.76	8.68	13.82	0.38	2.24	0.96	14.34	0.46	7.34	
	Bursa-PG5		2.25	12.26	15.96	949.43	0.60	11.74	11.21	3000.95	1.90	20.44	0.15	0.59	0.59	13.40	0.09	1.38	
	Bursa-PG6		2.79	10.53	19.93	1164.56	0.55	10.41	10.96	2681.15	2.35	11.23	0.26	0.66	0.52	14.76	0.04	0.83	
	Bursa-PG7		1.36	5.78	10.25	576.05	0.17	10.99	8.15	1776.03	0.71	10.06	0.38	1.16	0.56	6.54	0.40	1.37	
	Bursa-PG8		2.87	11.43	23.13	1246.89	1.04	11.43	4.88	1757.33	1.59	9.21	0.68	0.42	0.65	14.72	0.28	0.31	
	Bursa-PG9		3.09	10.11	35.54	1320.37	1.46	10.81	6.78	1527.40	1.82	10.34	0.71	0.73	0.40	11.96	0.46	0.83	
	Bursa-PG10		4.43	8.05	10.66	576.21	0.36	19.99	7.50	2715.86	6.32	24.76	0.38	2.81	1.04	12.81	0.16	0.80	
	Bursa-PG11		6.13	12.54	29.69	1329.39	1.35	12.55	10.23	2054.49	4.22	10.02	0.31	1.43	0.85	7.81	0.04	1.17	

na: not available

Table S3.I_{geo} and I_{geo} Class values of the HMs determined around the cement factory

HMs	PG1		PG2		PG3		PG4		PG5		PG6		PG7		PG8		PG10		PG11	
	I_{geo}	I_{geo} Class	I_{geo}	I_{geo} Class	I_{geo}	I_{geo} Class	I_{geo}	I_{geo} Class	I_{geo}	I_{geo} Class	I_{geo}	I_{geo} Class	I_{geo}	I_{geo} Class	I_{geo}	I_{geo} Class	I_{geo}	I_{geo} Class	I_{geo}	I_{geo} Class
**V**	0.141	1	-0.153	0	0.098	1	0.661	1	-1.043	0	-0.732	0	-1.769	0	-0.692	0	-0.065	0	0.403	1
**Cr**	1.644	2	0.909	1	0.718	1	2.515	3	-0.307	0	-0.526	0	-1.392	0	-0.408	0	-0.914	0	-0.274	0
**Mn**	-0.261	0	-0.561	0	-0.256	0	0.297	1	-1.740	0	-1.419	0	-2.379	0	-1.205	0	-2.322	0	-0.844	0
**Fe**	-0.154	0	0.252	1	0.482	1	1.047	2	-1.061	0	-0.766	6	-1.782	0	-0.668	0	-1.781	0	-0.575	0
**Co**	-0.546	0	-0.508	0	-0.635	0	0.064	1	-1.868	0	-1.993	0	-3.687	0	-1.074	0	-2.605	0	-0.698	0
**Ni**	0.661	1	0.371	1	0.183	1	0.861	1	-0.466	0	-0.639	0	-0.561	0	-0.505	0	0.302	1	-0.370	0
**Cu**	0.635	1	0.128	1	0.452	1	1.274	2	0.140	1	0.108	1	-0.319	0	-1.059	0	-0.439	0	0.008	0
**Zn**	0.269	1	-0.111	0	0.067	1	0.118	1	0.389	1	0.227	1	-0.367	0	-0.383	0	0.245	1	-0.157	1
**As**	1.134	2	0.639	1	1.003	2	1.669	2	-0.523	0	-0.216	0	-1.943	0	-0.780	0	1.211	2	0.628	0
**Sr**	-0.429	0	-0.559	0	-0.639	0	-0.166	0	0.398	1	-0.466	0	-0.625	0	-0.752	0	0.675	1	-0.630	1
**Cd**	-1.647	0	-1.877	0	-3.150	0	-1.487	0	-2.828	0	-2.034	0	-1.487	0	-0.647	0	-1.487	0	-1.781	0
**Sn**	0.609	1	0.045	1	0.520	1	1.033	2	-0.892	0	-0.730	0	0.083	1	-1.382	0	1.360	2	0.385	1
**Sb**	2.360	3	-0.585	0	0.379	1	0.678	1	-0.024	0	-0.206	0	-0.100	0	0.115	1	0.794	1	0.503	1
**Ba**	-0.529	0	-0.184	0	-0.201	0	-0.323	0	-0.421	0	-0.281	0	-1.456	0	-0.285	0	-0.486	0	-1.200	0
**Tl**	-0.494	0	-0.276	0	-1.154	0	-0.585	0	-2.939	0	-4.109	0	-0.787	0	-1.301	0	-2.109	0	-4.109	0
**Pb**	2.402	3	-0.115	0	-0.731	0	2.560	3	0.149	1	-0.585	0	0.138	1	-2.006	0	-0.638	0	-0.090	0

Table S4 Enrichment factor (EF) of the HMs

HMs	PG1	PG2	PG3	PG4	PG5	PG6	PG7	PG8	PG10	PG11
**V**	0.40	0.33	0.39	0.58	0.18	0.22	0.11	0.23	0.35	0.49
**Cr**	0.89	0.54	0.47	1.63	0.23	0.20	0.11	0.22	0.15	0.24
**Mn**	0.04	0.03	0.04	0.06	0.02	0.02	0.01	0.02	0.01	0.03
**Fe**	0.00	0.00	0.00	0.00	0.00	0.00	0.00	0.00	0.00	0.00
**Co**	1.03	1.05	0.97	1.57	0.41	0.38	0.12	0.71	0.25	0.92
**Ni**	1.02	0.83	0.73	1.17	0.47	0.41	0.44	0.45	0.79	0.50
**Cu**	2.24	1.57	1.97	3.48	1.59	1.55	1.15	0.69	1.06	1.45
**Zn**	0.67	0.51	0.58	0.60	0.73	0.65	0.43	0.42	0.66	0.50
**As**	46.46	32.97	42.43	67.33	14.74	18.23	5.51	12.33	49.02	32.73
**Sr**	0.08	0.07	0.07	0.10	0.14	0.08	0.07	0.06	0.17	0.07
**Cd**	114.93	98.03	40.56	128.45	50.70	87.89	128.45	229.86	128.45	104.79
**Sn**	2.39	1.62	2.24	3.20	0.84	0.94	1.66	0.60	4.02	2.04
**Sb**	616.00	80.00	156.00	192.00	118.00	104.00	112.00	130.00	208.00	170.00
**Ba**	0.04	0.05	0.05	0.05	0.05	0.05	0.02	0.05	0.04	0.03
**Tl**	49.16	57.19	31.10	46.15	9.03	4.01	40.13	28.09	16.05	4.01
**Pb**	12.86	2.25	1.47	14.34	2.70	1.62	2.68	0.61	1.56	2.29

Table S5 Ecological risk factor (Er_i) and potential ecological risk (RI) around the cement factory

HMs	Er_i									
	**PG1**	**PG2**	**PG3**	**PG4**	**PG5**	**PG6**	**PG7**	**PG8**	**PG10**	**PG11**
**V**	3.31	2.70	3.21	4.74	1.46	1.81	0.88	1.86	2.87	3.97
**Cr**	9.37	5.63	4.93	17.15	2.43	2.08	1.14	2.26	1.59	2.48
**Mn**	1.25	1.02	1.26	1.84	0.45	0.56	0.29	0.65	0.30	0.84
**Fe**	nc	nc	nc	nc	nc	nc	nc	nc	nc	nc
**Co**	5.14	5.27	4.83	7.84	2.05	1.88	0.58	3.56	1.23	4.62
**Ni**	11.86	9.70	8.52	13.62	5.43	4.81	5.08	5.29	9.25	5.80
**Cu**	11.64	8.19	10.26	18.13	8.27	8.08	6.01	3.60	5.53	7.54
**Zn**	1.81	1.39	1.57	1.63	1.96	1.76	1.16	1.15	1.78	1.35
**As**	32.91	23.35	30.05	47.69	10.44	12.91	3.90	8.74	34.73	23.19
**Sr**	nc	nc	nc	nc	nc	nc	nc	nc	nc	nc
**Cd**	14.37	12.25	5.07	16.06	6.34	10.99	16.06	28.73	16.06	13.10
**Sn**	nc	nc	nc	nc	nc	nc	nc	nc	nc	nc
**Sb**	nc	nc	nc	nc	nc	nc	nc	nc	nc	nc
**Ba**	nc	nc	nc	nc	nc	nc	nc	nc	nc	nc
**Tl**	10.65	12.39	6.74	10.00	1.96	0.87	8.70	6.09	3.48	0.87
**Pb**	39.64	6.93	4.52	44.22	8.31	5.00	8.25	1.87	4.82	7.05
$RI = \sum Er_i$	141.95	88.83	80.96	182.92	49.09	50.75	52.06	63.79	81.63	70.80

nc: not calculated
